# Common Questions and Misconceptions About Energy Drinks: What Does the Scientific Evidence Really Show?

**DOI:** 10.3390/nu17010067

**Published:** 2024-12-27

**Authors:** Jose Antonio, Brandi Antonio, Shawn M. Arent, Darren G. Candow, Guillermo Escalante, Cassandra Evans, Scott Forbes, David Fukuda, Maureen Gibbons, Patrick Harty, Andrew R. Jagim, Douglas S. Kalman, Chad M. Kerksick, Jennifer A. Kurtz, Joseph Lillis, Lonnie Lowery, Gianna F. Mastrofini, Scotty Mills, Michael Nelson, Flavia Pereira, Justin Roberts, Michael Sagner, Jeffrey Stout, Jaime Tartar, Adam Wells

**Affiliations:** 1 Department of Health and Human Performance, Nova Southeastern University, Davie, FL 33328, USA; 2 College of Health Professions and Sciences, University of Central Florida, Orlando, FL 32816, USAdavid.fukuda@ucf.edu (D.F.);; 3 Arnold School of Public Health, University of South Carolina, Columbia, SC 29208, USA; sarent@mailbox.sc.edu (S.M.A.);; 4 Faculty of Kinesiology and Health Studies, University of Regina, Regina, SK S4S 0A2, Canada; darren.candow@uregina.ca (D.G.C.); scottymills4@gmail.com (S.M.); 5 College of Natural Sciences, California State University, San Bernadino, CA 92407, USA; 6 Department of Physical Education Studies, Brandon University, Brandon, MB R7A 6A9, Canada; 7 Active Medical Solutions, The Woodlands, TX 77380, USA; 8 College of Science, Technology, and Health, Lindenwood University, St. Charles, MO 63301, USAckerksick@lindenwood.edu (C.M.K.); 9 Mayo Clinic Health System, La Crosse, WI 54601, USA; 10 Department of Psychology and Neuroscience, Nova Southeastern University, Davie, FL 33314, USAtartar@nova.edu (J.T.); 11 Department of Public Health & Exercise Science, Appalachian State University, Boone, NC 28607, USA; 12 Cambridge Centre for Sport & Exercise Sciences, Anglia Ruskin University, Cambridge CB1 1PT, UKjustin.roberts@aru.ac.uk (J.R.); 13 Walsh University, Department of Exercise Science, North Canton, OH 44720, USA; 14 Carrick Institute, Cape Canaveral, FL 32920, USA; 15 Keiser University, Fort Lauderdale, FL 33301, USA; fpereira@keiseruniversity.edu; 16 European Society of Preventive Medicine, Oxford Science Park, Robert Robinson Avenue, Oxford OX4 4GP, UK

**Keywords:** ergogenic aids, caffeine, supplements

## Abstract

Energy drinks are a commonly consumed beverage, and studies suggest a possible performance-enhancing effect. A Google Scholar search using the keywords “energy drinks” and “exercise” yields numerous results, underscoring the voluminous research on this topic. However, there are questions regarding the effectiveness and safety of energy drinks. These questions include, but are not limited to: (1) What are the main active ingredients in energy drinks? (2) Do energy drinks assist in weight management? (3) Do energy drinks enhance aerobic performance? (4) Do energy drinks enhance athletic speed? (5) Do energy drinks improve reaction time? (6) Do energy drinks enhance lean tissue mass? (7) Can energy drinks improve cognitive performance? (8) Does the acute consumption of energy drinks elevate resting energy expenditure? (9) Is there any evidence to suggest that energy drinks are more effective than an identical serving of caffeine alone? (10) Are there sex differences in the response to energy drink consumption? (11) Do energy drinks affect sleep or sleepiness? (12) Should pregnant women avoid energy drinks? (13) Do energy drinks adversely affect cardiovascular function? (14) Does consuming energy drinks cause brain damage? (15) What are other safety considerations regarding energy drinks? (16) Is there any evidence to suggest that energy drinks are more effective than an identical serving of caffeine alone? (17) If caffeine is the main active ingredient in energy drinks and coffee, why is there a discrepancy in the adverse events reported for each? To address these questions, we performed an evidence-based scientific evaluation of the literature on energy drink supplementation.

## 1. Introduction

Energy drink (ED) consumption has increased in popularity over the past few decades, primarily by those looking to improve resistance training capacity and/or performance in exercises commonly performed during resistance training sessions (i.e., bench press, squat). The main purported and most established ergogenic ingredient in commercially available EDs for improving measures of muscle performance is caffeine [1,2,3]. Caffeine, a trimethylxanthine, likely improves muscle performance (i.e., strength, endurance, power) [4,5] by acting as an adenosine-receptor antagonist [6,7], increasing calcium cycling in the sarcoplasmic reticulum [8], and/or by influencing plasma potassium, epinephrine, and phosphodiesterase levels [6,9,10].

Most commercially available EDs also contain taurine, a sulfonic amino acid compound found in skeletal muscle, which has been shown to improve muscle recovery and reduce muscle damage and fatigue [11]. Finally, some commercially available EDs contain carbohydrates, which may have beneficial effects on muscle performance [12]. Other common ingredients such as carnitine and the B vitamins likely play no significant role [13]. To address these questions, we conducted a narrative review of the literature to provide evidence-based answers to common misconceptions surrounding ED consumption. It should be noted that the format of this review is similar to previous publications by our research team [14,15].

## 2. What Are the Main Active Ingredients in Energy Drinks?

The recent evolution of the food and beverage industry has led to a robust increase in the variety of EDs sold each year and their ingredient profiles. EDs are classified as functional beverages with purported benefits, including increased energy, improved focus, and increased metabolism while mitigating the onset of fatigue [16]. Because of the diversity of products available on the market, it is difficult to delineate the specific ingredient profile of these types of beverages, as foods such as carbonated flavored waters and sports drinks now often have caffeine, electrolytes, or other active ingredients. But traditionally, EDs are ready-to-drink beverages containing 8–16 fl oz. and contain caffeine, B vitamins, electrolytes, and other botanicals or nootropic ingredients [16]. Energy shots are a similar category of functional beverages, just presented in smaller, more concentrated formulations (~3 fl oz.) [16].

In a recent article, the top 75 selling commercially available EDs in the United States (as of 2021) were identified and used to help establish a profile of the most prevalent ingredients found in EDs and compile an ingredient summary, as seen in Table 1. The list provides the prevalence of the most common ingredients and quantities (if known). Caffeine was found to be the most prevalent ingredient in ED. Its stimulatory actions serve as a foundational ingredient and the desired effect of ED, hence the name of the products themselves. Some EDs are formulated in a way to be stimulant-free; however, they likely do not elicit the same effects in terms of their ability to increase alertness, cognitive function, and memory recall, in addition to some of the physical performance-related benefits that are well-documented from caffeine-focused research [2]. There are some concerns regarding the caffeine content of EDs, with claims of excessive amounts of caffeine contained per serving. However, the average caffeine content of 174 mg per serving commonly found in EDs (Table 1), is comparable to two servings (or cups) of an 8 oz brewed drip coffee and in alignment with other caffeine-containing products (Table 2). Additionally, the caffeine content of EDs would be considered safe as 174 mg of caffeine is well below the 400 mg threshold established by the Food and Drug Administration, which has been proposed as an upper limit to avoid adverse effects [17]. Sugar was found to be another common ingredient, found in ~45% of EDs; however, it’s worth noting that several companies often manufacture a carbohydrate-containing beverage as well as a sugar-free or low-calorie option, depending on the consumer’s preference. In addition to caffeine and sugar, B vitamins and electrolytes were the next most prevalent ingredients, with 35–75% of EDs containing varying amounts of these ingredients, all of which can have important implications for overall health (i.e., metabolism and immune support) if found to be low in the diet or because of excessive sweating [17].

Several ingredients commonly found in EDs have been shown to elicit favorable outcomes regarding cognitive and physical function, as well as overall health when consumed individually, as outlined in a 2023 Position Stand on EDs published by the International Society of Sports Nutrition [17]. In addition to caffeine, taurine has also been shown to have a positive effect on hormone function and metabolism. It also serves as an anti-inflammatory agent with antioxidant properties, likely resulting in a neuroprotective effect [18,19,20]. The combination of the aforementioned ingredients may offer a synergistic benefit, depending on the specific dosages found in each beverage. For a complete list and description of the more common ingredients, readers are directed to the previously mentioned publications [16,17] which provides a more in-depth discussion of the mechanisms of action and any documented health and performance benefits.

**Table 2:** Caffeine content per serving of caffeine-containing products. Reproduced from Jagim et al. [17], which is licensed under an open-access Creative Commons CC BY 4.0 license.

Beverage	Caffeine (mg)
Energy Drinks [16]	160
Red Bull© (12 oz.)	111
BANG© (16 oz.)	300
Coffee (8 oz.)	90
Commercial Coffee (20 oz.)	410
Green Tea (16.9 oz.)	94
Soda (12 oz.)	35
Pre-Workout Supplements [21]	254
Caffeine Capsule	200

Oz = fluid ounces.

In summary, caffeine, sugar, taurine, and B-vitamins are several of the more common ingredients found in energy drinks. Caffeine is likely to play the most important role regarding the ergogenic effects of energy drinks.

## 3. Do Energy Drinks Assist in Weight Management?

Energy drink formulations commonly include ingredients like caffeine and green tea extract, which have been shown to increase how many calories someone expends as well as increase the levels of glycerol and free fatty acids found in the blood [22]. Beyond this, several published research studies have examined the ability of an energy drink to impact calorie-burning rates [23,24,25,26], fat oxidation, and blood-based markers of lipolysis. While these findings offer excitement and reasons to suggest that daily consumption of an energy drink may favorably impact weight loss and weight management, one must temper this excitement and realize that nearly all these studies examined changes after just one dose of consumption. Weight loss occurs over a sustained period where energy expenditure rates exceed energy intake. As such, the best evidence to evaluate whether an energy drink can impact weight loss and weight management should come from studies completed over an extended period.

Following these guidelines, a limited number of studies have evaluated whether energy drink consumption beyond one day can impact weight loss or weight maintenance. In 2008, Roberts et al. [27] assigned 60 healthy, college-aged males and females in a single-blind manner to consume either a carbonated, energy drink (Celsius, Boca Raton, FL, USA) or a carbonated, caffeine-free, commercially available diet soda (Caffeine-Free Diet Coke, Coca Cola, Inc., Atlanta, GA, USA) for 28 days. The energy drink delivered 10 kcals, 200 mg of caffeine (approximately 2.8 mg∙kg^−1^) and a proprietary blend of guarana extract (caffeine source), green tea, glucuronolactone, ginger extract, and taurine. Changes in body composition were evaluated before and after 28 days of ingestion while acute changes in resting metabolic rate and markers of lipolysis secondary to drink ingestion were also evaluated on day 0 and day 28 of ingestion. After 28 days of ingestion, energy drink consumers experienced statistically greater decreases in percent body fat and fat mass when compared to the control group while body mass changes tended to be different between the two groups. In addition, free fatty acid levels were measured in the blood and energy drink consumption significantly increased (*p* < 0.05) free fatty acid levels greater than the control group. These initial results were followed up with a sex-based analysis that revealed women had higher free fatty acid levels when compared to men, however, males who ingested the energy drink lost significantly more body fat [24].

A longer, more comprehensive study by Lockwood et al. [28] examined the impact of consuming an energy drink for ten weeks with and without an exercise program. Sedentary males (*n* = 38) were assigned to one of four groups (exercise vs. no exercise and energy drink vs. placebo) and were evaluated for changes in body composition. Just consuming the energy drink without exercise did not impact any changes in body composition, but when a daily energy drink was combined with a weekly exercise program, greater improvements in body composition were observed when compared to those people who followed the same exercise program but consumed a placebo. From the same study, Smith and colleagues [29] published a paper that included 27 women and concluded that energy drink consumption over ten weeks with or without an exercise program did not impact the observed changes in body composition. Finally, a 4-week study was published by Seidler et al. [30] where they assigned 52 healthy, exercise-trained, young adults to consume a protein supplement with and without daily consumption of an energy drink or a control group, which consumed no supplement at all. As with the Roberts and Dalbo studies, no exercise or diet changes were required. While the combination of protein and energy drink did increase resting energy expenditure, no changes in body composition or body mass were identified in this study.

Very limited research exists to highlight any impact that an energy drink may have on body mass or body composition. While results from one study [27] do offer preliminary support for the potential for energy drinks to impact weight management or body composition studies, other studies (Lockwood 2010, Smith 2010, Seidler 2023) failed to reach similar conclusions [28,29,30]. The Lockwood and Smith studies do provide support that consuming an energy drink as part of a greater exercise program can support positive changes in fitness and body composition, but these results are routinely observed from exercise alone which brings into question what impact, if any, the energy drink had on body mass changes. More research needs to be completed to better understand if daily consumption of an energy drink for several weeks to months with and without prescribed diet and exercise exerts any additional impact on body mass or body composition.

In summary, there is a scarcity of data on the effects of chronically consuming energy drinks on body composition. Thus, it is reasonable to conclude that they have no impact on body composition.

## 4. Do Energy Drinks Enhance Aerobic Performance?

Many studies have reported positive effects of EDs on various measures of endurance performance [17]. These effects have been observed in different sports and exercise protocols. For example, during cycling, Ivy et al. [31] found that consuming an ED containing 160 mg of caffeine, taurine, glucuronolactone, and carbohydrate 40 min before exercise improved time-trial performance by 4.7% compared to placebo. Similarly, Geib et al. [32] observed that endurance athletes who consumed the same ED containing 160 mg of caffeine and 54 g of carbohydrate showed significantly longer time-to-exhaustion during a cycling protocol than a placebo. The benefits of EDs extend to team sports. Del Coso et al. [33] found that soccer players who ingested an ED covered more distance at speeds higher than 13 km · h^−1^ during a simulated match compared to placebo. Prins et al. [34] reported a 2.8% improvement in 5 km running time after participants consumed a similar ED containing 160 mg of caffeine and 54 g of carbohydrates compared to a placebo.

Furthermore, positive effects of EDs have been observed in various sports. Del Coso et al. [35] conducted a series of studies examining the effects of EDs that provided 3 mg/kg body weight of caffeine. They found improvements in performance metrics for soccer, rugby, field hockey, and tennis players, including greater distances covered at high intensities, increased running speeds, and better overall performance during simulated competitions. However, not all studies have found significant performance benefits from ED consumption. Sanders et al. [36] observed no improvement in oxygen consumption or perceived exertion during treadmill running with various EDs compared to a placebo. Similarly, Candow et al. [37] reported no improvement in running time to exhaustion with Red Bull ED versus a placebo drink. These conflicting results highlight the complexity of the topic and suggest that various factors may influence the effects of EDs.

Several key factors appear to influence the effectiveness of EDs on aerobic performance. Studies show positive effects typically used ED doses containing ≥3 mg/kg of body weight caffeine [17,33,34]. Lower doses may not be as effective in obtaining performance benefits. Talanian and Spriet [38] demonstrated this dose-response relationship. They found that a moderate dose of caffeine (200 mg or 2.9 mg/kg body weight) improved cycling time trial performance more than a low dose (100 mg or 1.5 mg/kg body weight), and both doses were more effective than the placebo. However, Alford et al. [39] found performance benefits even when consuming half the typical serving size of Red Bull ED, suggesting that lower doses may still be effective in some cases.

The timing of ED consumption appears to be important. Most studies administered EDs 30–60 min before exercise, allowing for peak caffeine absorption. This timing strategy aligns with the pharmacokinetics of caffeine, which typically reach peak plasma concentrations within 30–60 min after ingestion [31,33]. Interestingly, Talanian and Spriet [38] found performance benefits even when caffeine was ingested later in exercise, specifically at 80 min in a 120-min cycling protocol, suggesting that caffeine can be effective when consumed during prolonged exercise.

The duration and nature of the exercise task may also influence the efficacy of EDs. Benefits seem more pronounced in longer-duration activities, such as hour-long cycling time trials or 5 km runs, and various investigations have found improvements in repeated sprint ability and high-intensity running during a 90-min simulated soccer match, but the effects were less consistent [31,33,34]. This difference may be due to the time required for caffeine to fully exert its physiological effects as well as the increased relevance of factors such as glycogen sparing in longer events.

Individual responses to EDs can vary significantly, partly because of the genetic factors that influence caffeine metabolism. Womack et al. [40] found that individuals with a certain variant of CYP1A2 (a gene responsible for caffeine metabolism), specifically AA homozygotes, experienced greater ergogenic effects of caffeine than did carriers of the C allele. This genetic variation may explain some of the inconsistencies observed between studies and highlights the importance of considering individual factors when evaluating the potential benefits of EDs.

Regular caffeine consumption may lead to tolerance, potentially reducing the acute effects of ED consumption [27]. However, research on this topic has been inconclusive. Gonçalves et al. [41] found that habitual caffeine intake did not significantly influence the ergogenic effect of caffeine on cycling performance, suggesting that EDS may be effective, even for regular caffeine consumers.

The combination of ingredients in EDs makes it difficult to isolate the effects of the individual components. Although caffeine is likely the main ergogenic ingredient, other substances may also contribute to the overall effect. For example, taurine may enhance calcium handling in the muscle cells, potentially improving contractility [17]. However, few studies have successfully isolated the effects of these individual ingredients in the context of ED consumption and aerobic performance [17].

In summary, the available evidence suggests that energy drinks may provide modest benefits to endurance performance, particularly for longer-duration activities. These effects are likely primarily due to caffeine content, although other ingredients may also play a role. Although some studies have shown improvements in the 2–5% range in performance measures, others have not found significant benefits.

## 5. Do Energy Drinks Enhance Athletic Speed?

Even though commercial EDs may contain several nutrients (e.g., taurine, L-carnitine, sodium bicarbonate) that may have ergogenic properties, the scientific evaluation of EDs on athletic speed predominantly, and unsurprisingly, focuses on caffeine content. Most research has targeted team sports, with a comparative determination that the relative dose of caffeine in EDs is a requisite for any ergogenic effect on acute athletic speed. By definition, this indicates that the total volume of product consumed is also essential.

Del Coso and colleagues [33,35,42,43,44,45,46] for example, have undertaken several studies investigating commercial ED formulas (e.g., Red Bull [sugar free], and Fure^®^, ProEnergetics) employing a ~3 mg·kg^−1^ caffeine dose. When compared to a placebo control, the inclusion of caffeinated ED surrounding exercise significantly increased mean peak sprint speed (26.3 ± 1.8 km·h^−1^ v 25.6 ± 2.1 km· h^−1^, *effect size [ES]: 0.33*) during a repeated sprint test, and increased number of sprints (30 ± 10 v 24 ± 8, *ES*: 0.75) and distance covered at sprint speed (>18 km·h^−1^; *ES:* 0.51) during a simulated soccer match [33]. Similar findings have been observed in female soccer [44], field hockey [42], men’s rugby players [35], elite junior tennis players, and 19 elite junior tennis players, although the authors do raise the potential limitation of motivation when conducting controlled testing. However, the increased instantaneous speed observed in these studies during match-play demonstrates that the ‘pace of movement patterns’, particularly at sprint speeds, could impart a physical advantage in these sports (increasing the running distance covered by ~13–34%).

In contrast, in women’s rugby sevens [43], the same ED did not impact repeated sprint assessment (6 × 30 m) or maximal running speed during match-play but did increase the pace at sprint velocity (4.6 ± 3.3 v 6.1 ± 3.4 m∙min^−1^), demonstrating a partial effect of the caffeinated ED. Elsewhere, a low caffeine intake via consumption of a 250 mL Red Bull (containing 80 mg caffeine (relative: 1.3 mg·kg^−1^ body mass [BM])), 1 h pre-exercise) had no impact on repeated sprint time in female soccer players [47]. Whilst more research is needed, these findings highlight both dose and gender-specific effects of EDs [48], with female athletes potentially requiring >3 mg·kg^−1^ caffeine to elicit ergogenic effects.

For individual sports, the use of EDs has been shown to enhance the time to complete a 50 m simulated swim [46] with a suggestion, adding caffeine enhances central drive via adenosine receptor interaction and may impact anaerobic glycolysis [49]. When extended to sustained speed efforts, Candow and colleagues reported no effect of a caffeinated ED (sugar-free Red Bull, 2 mg∙kg^−1^ caffeine) on high-intensity run time to exhaustion [37]. Elsewhere, the use of a non-caffeinated ED (containing Calamansi juice [vitamin C], taurine and glucose) did not impact 3 km run speed (2.34 ± 0.30 m∙s^−1^ v 2.32 ± 0.30 m∙s^−1^, *p* > 0.05) [50], again inferring that caffeine content (and relative dose) are key determinants of any ergogenic effect on athletic speed. Further supporting this, the use of varied relative doses of caffeinated EDs (500 mL Red Bull, caffeine range: 1.5–3.9 mg·kg^−1^, mean: 2.9 mg·kg^−1^) resulted in 78% of participants completing a 5 km run faster than placebo [51] and, although speed was not directly quantified, this highlights a ~2% improvement in performance. However, it should be noted that the placebo did not include other potentially ergogenic nutrients (e.g., taurine, proposed to regulate intracellular Ca^2+^ handling [52]) found in the ED. It is also noted that higher intakes may be needed for individuals already caffeine-habituated [53] and not all studies support the use of EDs for athletic speed or repetitive sprints [52], including when consumed as lower caffeine doses or ‘energy shots’ [54] prior to sustained aerobic efforts. Additionally, despite the successful marketing and promotion of EDs in a sporting context, it is important that overuse is avoided to help mitigate possible short- and long-term side effects [55]. Short-term effects such as headaches and insomnia are most frequently reported. However, longer-term effects that may damage cardiovascular and neurological systems are a risk if EDs are frequently or excessively used [56].

In summary, based on the available evidence, the use of strategic caffeinated EDs at a relative dose >3 mg·kg^−1^ demonstrates evidence of enhanced athletic speed in individual and team sports, irrespective of gender, supporting previous position statements [17].

## 6. Do Energy Drinks Improve Reaction Time?

EDs are purported to improve reaction time. Their primary ingredient is caffeine, which acts as an adenosine receptor antagonist, increasing arousal, motivation, and motor responses [57]. Although mixed, a growing body of evidence demonstrates the effectiveness of EDs in various populations, as reviewed by Jagim et al. [17]. For example, Alford et al. [39] employed a crossover randomized placebo control design where participants (*n* = 36, young males and females) received either a placebo or an ED containing 80 mg of caffeine. There was a significant improvement 30 min following the ingestion of the ED, while no change was found for the placebo on a 5-choice reaction time test. Goel et al. [58] found similar improvements in auditory reaction time with a relative dose of caffeine 2 mg/kg provided in an ED in 20 medical students (10 males and 10 females) when ingested 60 min before testing. Furthermore, they noted that both males and females demonstrated similar improvements. These findings have also been replicated using sugar-free ED. For example, Concerto et al. [59] employed a randomized crossover design in 14 healthy adults to determine the effects of a sugar-free ED and revealed significant improvements in reaction time. Howard and Marczinski [60] examined whether the dose altered the effects on reaction time. Eighty young adults were randomized to one of five conditions: a low dose (1.8 mL/kg energy drink), a moderate dose (3.6 mL/kg), a high dose (5.4 mL/kg energy drink), a placebo, or a no drink condition. Overall, the ED, independent of dose, improved reaction time compared to placebo; interestingly, the lowest dose revealed the greatest improvements.

In contrast, others have found no improvements [17,61,62,63]. For example, in a randomized, double-blind, placebo-control cross-over counterbalanced trial, Antonio et al. [61] found no benefits of an ED containing 300 mg of caffeine on reaction time in exercised-trained adults. Howden et al. [62] found that an ED containing caffeine did not affect brake reaction time versus a placebo. Also, Evans et al. [63] found no effect of an ED on reaction time. Similarly, Pereira et al. [64] examined whether an ED (Gorilla Mind™, Boise, ID, USA) had greater effects on sustained attention, mood, handgrip strength, and push-up performance than a caffeine-matched control drink in exercise-trained participants. While the ED group showed improved reaction time, the difference between the ED and caffeine control was not statistically significant. These results suggest that the additional ingredients in the ED may not provide notable benefits beyond caffeine for these outcomes in active individuals. It is important to note that the caffeine dose was, on average, less than 3.0 mg/kg body weight, which is likely an inadequate dose.

In summary, there is evidence that energy drinks containing caffeine can improve visual and auditory reaction time, although results are mixed. It should be noted that a dose of caffeine less than 3 mg per kg body weight is usually insufficient to improve reaction time.

## 7. Do Energy Drinks Enhance Lean Tissue Mass?

From a lean tissue mass perspective, there is some evidence in rodents that caffeine can increase ribosomal S6, a protein kinase involved in the mammalian target of rapamycin (mTOR) signaling pathway, which governs muscle accretion. However, caffeine did not influence other kinases such as 4E-BP1, p70S6K, or plantaris muscle growth [65]. In the only study examining the effects of ED consumption during an exercise training program, Lockwood et al. [66] showed that the daily ingestion of a commercially available ED (containing 200 mg of total caffeine) for ten weeks did not augment measures of lean tissue mass or muscle strength (compared to placebo) following a multi-modal training program which included resistance training (2 sessions/week; 9 whole-body exercises; 1 set of 8–12 repetitions) in young, healthy adults (23–26 yrs). Independent of resistance training, there is some evidence that commercially available EDs can improve measures of muscle performance in exercises routinely performed during training sessions. For example, Forbes et al. [13] showed that the consumption of a commercially available ED (containing 2 mg of caffeine/kg body mass) significantly improved bench-press muscle endurance (as measured across 3 sets of repetitions performed to volitional fatigue using 70% baseline 1-repetition maximum) by two additional repetitions compared to placebo in young, healthy adults (21–26 yrs). Similarly, Astley et al. [67] showed that the ingestion of a commercially available ED (containing 2.5 mg of caffeine/kg body mass) increased the number of repetitions performed to fatigue for the bench press (+2 repetitions) and knee extension (+2 repetitions) exercises in young males (21 yrs) compared to placebo. Finally, Del Coso et al. [68] showed that the ingestion of a commercially available ED (containing 3 mg of caffeine/kg body mass) significantly increased maximal power output during the half-squat (+172 Watts) and bench press (+26 Watts) exercises in young adults (30–37 yrs) compared to placebo. Interestingly, consuming the ED containing only 1 mg of caffeine/kg body mass did not influence power output in these exercises. In contrast, Eckerson et al. (2013) found no muscle benefits from the consumption of a commercially available ED (containing 160 mg of caffeine) 1-h before performing tests of maximal strength (1-RM) and endurance (repetitions to volitional fatigue using 70% baseline 1-RM) for the bench press exercise in young males (19–25 yrs) compared to placebo.

In summary, there is evidence that consuming commercially available energy drinks can improve upper and lower body muscle endurance and power in young healthy adults. However, there is no evidence that energy drinks can increase lean tissue mass in humans.

## 8. Can Energy Drinks Improve Cognitive Performance?

The effect of several commercially available EDs or shots on cognitive performance has previously been evaluated in placebo-controlled trials, the most extensive being the Red Bull^®^ ED. An absolute dose of Red Bull^®^ ED (250 mL, 80 mg caffeine) has been shown to significantly improve subjective alertness scores, concentration and immediate recall memory [39], and to improve driving performance (decreased lane drifting and improved steering control) along with subjective feelings of alertness and fatigue during a prolonged driving task (3–4 h) [69]. Superior composite measures of response accuracy and information retrieval speed within working memory and episodic memory tasks have also been observed compared to equivalent Sugarfree Red Bull^®^ and placebo [70]. A larger absolute dose of Red Bull^®^ (500 mL, 160 mg caffeine) was shown to improve, immediate memory, verbal fluency, attention, and performance in a mental stress test 30-min post-ingestion [71]. Relative doses of Red Bull^®^ and Sugarfree Red Bull^®^ (ranging from 1.8 to 5.4 mL/kg) have shown varying responses in reaction time [58,59,60] but have yet to be fully explored with regard to other cognitive domains. A minority of other commercially available EDs have also shown negative results. Reload™ ED (Oakland, CA, USA) (approx. 200 mg caffeine) did not influence attention or working memory when compared to placebo in nine elite eSports players during 3 competitive League of Legends games beginning 30-min post-ingestion, although caffeine alone was also not beneficial when compared to placebo [72]. Similarly, Redline™ ED (355 mL, 300 mg caffeine) did not improve inhibitory control, selective attention or cognitive flexibility (executive function) 30–45 min following ingestion when compared to placebo drink, despite improvements in pattern comparison processing speed and fewer false starts in the psychomotor vigilance test following the ED [63].

Energy shots are also a large category of energizing products, largely dominated by the 5-h Energy Shot^®^ for which there are also several examinations of cognitive effects. One study showed significant improvements in 6 validated composite measures of cognitive function generated from 10 tests administered via the Cognitive Drug Research (CDR) test battery, including power & continuity of attention, quality of working and episodic memory, speed of memory and reaction time variability, the majority of which persisted up to 6-h post-ingestion of the 5-h Energy^®^ shot. In contrast, 5-h Energy^®^ did not significantly improve short- or long-term cognitive function for selected computer-based tasks administered every hour for 5 h post-ingestion, despite a high level of perception that it was working effectively compared to a placebo [73]. Similarly, 5-h Energy Shot^®^ did not improve subjective measures of energy, mood or cognitive performance in Trail Making or Digit Symbol Substitution tests, although 200 mg caffeine was also ineffective when compared to placebo [74]. The 5-h Energy^®^ Shot also failed to influence behavioral control (cued go/no-go task), despite improvements in subjective states of vigor and fatigue, while a decaffeinated version of the 5-h Energy^®^ shot also had no effect on mood, visual information processing, or distraction avoidance compared to placebo 30-min, 2.5 and 5-h after ingestion [75].

The most common component of EDs is caffeine, which is thought to mediate many of the observed beneficial effects of EDs and shots. However, other potentially ergogenic ingredients may extend the beneficial effects of caffeine on cognition [17,76]. Notably, Red Bull^®^ ED has been shown to be more effective than Sugarfree Red Bull^®^ in improving cognitive performance [70], with the only difference between the two being the glucose content. A study by Scholey and Kennedy [77] showed similar findings in that neither glucose or caffeine in isolation significantly improved measures of mood or cognition assessed via the same CDR battery utilized by Buckenmeyer [73], whereas the combination of ingredients provided significant improvements in secondary memory and accuracy of attention, without disturbing mood. Adan and Serra-Grabulosa [78] also showed that the combination of 75 mg caffeine and 75 g of glucose had beneficial effects on learning and consolidation of verbal memory, as well as attention tasks that were not apparent when administered alone, while Giles et al. [79] showed that 200 mg caffeine combined with 50 g glucose enhanced working memory. Others have reported report possible minor effects of carbohydrates on cognitive measures when added to caffeine [80,81]. The potential synergistic effects of caffeine and glucose are also noted in several reviews [3,82], although in general, the evidence appears to be weak due in part to a paucity of dose-response studies and a lack of research examining both the combination and isolation of each ingredient [83].

Other potentially ergogenic combinations of energy drink/shot ingredients include caffeine and taurine [84,85], and L-theanine [86,87]. Evidence for the synergistic effects between caffeine and other ingredients, however, is either limited or absent entirely [82]. It is noteworthy to mention that many prior studies include a period of caffeine abstinence before the administration of ED or energy shot interventions, raising the question as to whether the observed effects of EDs and shots on cognition reflect a true ergogenic effect or simply a reversal of the adverse effects associated with caffeine withdrawal. Overall, there is a need for more well-designed, randomized, placebo-controlled studies to better assess the claims of improved cognition made for the non-caffeine components of EDs. Furthermore, situations where cognitive impairments are associated with sleep deprivation [70] or fatigue may be an area of interest for future investigation.

In summary, several studies have evaluated the effects of energy drinks on cognitive performance, showing improved alertness, concentration, memory, and attention. Caffeine is believed to drive many benefits, but the combined effects of other ingredients like glucose and taurine need further investigation, and more well-designed studies are necessary.

## 9. Does the Acute Consumption of Energy Drinks Elevate Resting Energy Expenditure?

Various studies from different labs have investigated the impact of acute ED consumption on resting energy expenditure (REE) [24,25,26,68,88,89,90]. In five of the seven studies, the acute consumption of various EDs significantly increased REE compared to a placebo [25,26,88,89]. In contrast, two studies reported no significant differences in REE with the acute consumption of an ED [24,68]. In one of the first studies that investigated the metabolic responses to the acute consumption of an ED [88], 20 healthy men and women were randomly assigned to ingest 12 ounces of the Celsius™ ED or caffeinated diet soda on separate days using a randomized, counterbalanced design. REE was measured at baseline before the ingestion of either product and at the end of each hour for 3 h post-ingestion. REE increased by 13.8% after 1 h, 14.4% after 2 h, and 8.5% after 3 h of ingesting Celsius™. On the other hand, the diet soda intake did not significantly increase REE. Interestingly, in a larger 2010 study [24], researchers had 60 participants consume a Celsius™ ED in a single-blind, matched-pairs, placebo controlled study. REE was assessed before and 60-, 120-, and 180-min post consumption of the ED or a noncaloric and non-caffeinated placebo, but no significant increases in REE were reported.

In a study that investigated the acute metabolic responses of consuming a different ED, Rashti and colleagues [26] looked at the effects of the Meltdown™ ED on healthy and physically active women. Ten women participated in two testing sessions performed in a randomized and double-blind fashion. The participants reported to the laboratory after a 3 h fast and were provided either with the ED or a placebo, and REE was measured every 5 min during the first 30 min and every 10 min during the next 150 min. Although no difference in REE was seen in the first hour, significant differences were reported in the second and third hours, with a 13.9% increase and an 11.9% increase, respectively.

In another study, Clark and colleagues [90] had 32 recreationally active caffeine consuming participants volunteer to complete three visits in a randomized, double-blind, crossover fashion. Each visit began with a baseline measurement of REE and was followed by the ingestion of an ED containing 140 mg or 100 mg of caffeine or a placebo. The REE measurements were repeated at 30, 60, 90 min post-ingestion, and the researchers reported an increase in total REE estimated from an area under the curve analysis for the 140 mg caffeine formula compared to the 100 mg caffeine formula and the placebo. Furthermore, REE also increased in the 100 mg caffeine formula as compared to the placebo. Within the first 60 min post-ingestion, the 140 mg of caffeine formula increased REE by 5.8%, but the 100 mg formula increased REE by only 3.9%.

Recently, investigators recruited 28 male and female volunteers to complete two visits in a randomized, double-blind, crossover fashion [89]. REE was measured at baseline for each visit, which was then followed by consumption of an ED (OxyShred Ultra Energy, 180 mg caffeine) or a placebo (PL). REE measurements were repeated at the intervals of 35–50- and 85–100-min post-consumption, and researchers reported higher REE values at the 35–50 min time frame (5.2% higher) and 85–100 min time frame (5.5% higher) after the consumption of the ED as compared to PL.

In contrast to most of the studies that reported increases in REE after the acute consumption of an ED, Del Coso et al. [68] examined the effects of three doses (0 mg/kg vs. 1 mg/kg vs. 3 mg/kg) of a caffeine-containing ED (Fure^®^) in a double-blind, placebo-controlled, randomized, and repeated measures experimental design. Interestingly, no differences in REE were reported with the ED, regardless of the caffeine content. However, it should be noted that participants for this study were instructed to consume a light meal for at least 2 h before the experimental trials, and this may have influenced the results since food consumption can increase REE. As such, the natural increase in REE from food consumption may have masked the increase in REE that would have been seen from the ED.

In summary, various commercially available energy drinks can acutely increase REE for up to 3 h after ingestion. Moreover, current evidence that aligns with other research suggests a direct positive relationship between the quantity of caffeine in an energy drink and its effect on REE [90].

## 10. Are There Sex Differences in the Response to Energy Drink Consumption?

The current body of evidence shows that EDs provide ergogenic benefits regardless of sex [17]. To date, a wide variety of investigations have reported performance, cognitive, and metabolic outcomes following ED consumption in samples consisting of males only [42,46,91,92,93], females only [43,44,47,94,95], or both [13,23,24,25,31,39,51,68,70,96,97]. The absence of major sex differences resulting from ED consumption is somewhat unsurprising, as caffeine is a central ingredient in best-selling ED and energy shot product formulations [16] and has been shown to have minimal sex differences in its acute ergogenic potential [2]. However, it should be noted that nearly all studies investigating ED using mixed-sex cohorts did not directly examine sex differences after ED consumption but rather computed general changes in outcome variables across their entire samples.

The metabolic potential of ED consumption has been well-demonstrated across multiple studies in both male and female participants. For example, Dalbo and colleagues [23] showed consistent increases in resting energy expenditure after ED consumption in 30 male and 30 female participants. A later study in a similar population by the same research group [27] found that male and female participants who consumed an ED product for 28 days experienced significant reductions in body fat percentage and fat mass compared to placebo. Harty et al. [25] likewise demonstrated significant acute increases in resting metabolism relative to placebo after consuming a protein-containing ED product in both active males and females. However, it should be noted that Dalbo and colleagues [24] conducted a later study in 30 male and 30 female participants, finding that males lost significantly more body fat percentage compared to females across 28 days of ED use, among other metabolic differences. Nonetheless, the metabolic impact of energy products appears relatively consistent across the sexes.

To date, a variety of studies have shown the ergogenic impact of ED consumption on sport-related outcomes in both males and females—including simulated soccer performance [33], volleyball-specific jump and spike testing in male volleyball athletes [91], running pace and power output during repeated jump testing in female rugby athletes [43], total distance covered during a simulated game in elite male field hockey players [42], and countermovement jump height and sprinting performance in female soccer players [44]. Likewise, Forbes and colleagues demonstrated improvements in total bench press muscular endurance after consumption of an ED in a mixed-sex cohort [13]. Prins et al. [51] also reported beneficial findings in an endurance exercise model, showing that ED consumption increased 5-km running performance in male and female recreational endurance runners. Ivy and colleagues [31] likewise recruited six male and six female cyclists to consume an ED or placebo before cycle time-trial testing, reporting that performance was enhanced in the supplementation group. Interestingly, Salinero and colleagues [98] also found that subjective self-perceptions of physical performance and the prevalence of reported side effects were similar in male and female participants following the consumption of an ED product.

In summary, though there is limited evidence to suggest the presence of sex differences in response to energy drinks, the preponderance of evidence demonstrates that energy drinks do indeed have an ergogenic effect in both males and females.

## 11. Do Energy Drinks Affect Sleep or Sleepiness?

EDs are commonly used to increase wakefulness, improve alertness, and enhance physical performance. The mechanisms through which these changes occur are mostly through the effects of caffeine on the nervous system. While ED often contain other ingredients such as taurine, guaraná, ginseng, glucuronolactone, B-vitamins, and other compounds, the general effects of EDs on alertness, cognitive functioning and sleep are primarily due to their caffeine content [99].

Caffeine’s impact on cognition is well-documented and is related to the effect of caffeine on mental alertness. Indeed, there is a general scientific consensus that caffeine enhances “lower” cognitive functions, such as simple reaction time, attention, and vigilance. Studies suggest that caffeine doses ranging from 32 to 300 mg (approximately 0.5–4 mg/kg for a 75 kg individual) significantly improve basic cognitive tasks, including reducing response times and error rates in simple reaction time tasks, choice reaction time tasks, and visual vigilance tasks [100,101]. Additionally, caffeine has been shown to positively influence mental alertness/vigilance [102,103,104,105]. It is important to note that the effects of caffeine on “higher” cognitive functions, such as problem-solving and decision-making, remain uncertain [106,107]. Some research indicates that caffeine can improve executive functions, such as task switching and visual selective attention, though these effects are more variable and dose-dependent [108,109,110]. Notably, also, high doses of caffeine that are found in EDs may increase tension, anxiety, and jitteriness, potentially negating their cognitive benefits [111,112]. The classic inverted U-shaped arousal-performance function, as described by the Yerkes-Dodson law, suggests that while ED use may enhance cognitive performance by increasing arousal, excessive arousal due to sleep deprivation or high caffeine intake from ED can ultimately degrade cognitive function [113].

The underlying mechanism through which caffeine can increase alertness also relates to its effects on increasing wakefulness and impairing sleep. A main mechanism involves caffeine’s antagonistic action at adenosine A1 and A2A receptors—especially in dopamine-rich brain areas, which increases dopaminergic activity, resulting in heightened wakefulness and motor activity [114,115,116]. Peak plasma concentrations of caffeine occur 30 min after ingestion, aligning with the rapid onset of its cognitive effects [117]. The caffeine in ED results in antagonism of adenosine receptors, particularly A1 receptors, which are abundant in brain regions such as the hippocampus, cortex, cerebellum, and hypothalamus, disrupts the homeostatic sleep drive [118,119,120]. Adenosine plays a crucial role in promoting sleep, and by blocking its action, caffeine delays sleep onset, reduces total sleep time, and impairs sleep quality. This disruption is particularly pronounced when caffeine is consumed in the afternoon or evening, as the stimulant’s half-life can extend for 5–6 h (Nehlig, 2010) [110].

In addition to delaying sleep onset and reducing total sleep time, caffeine consumption has been shown to decrease the proportion of deep sleep stages, particularly slow-wave sleep, which is critical for restorative rest and cognitive function [121,122]. This reduction in deep sleep can lead to a cumulative sleep debt over time, especially in individuals who habitually consume EDs late in the day [123]. Moreover, caffeine’s disruption of sleep architecture often results in increased sleep fragmentation, causing frequent awakenings and reducing overall sleep efficiency [124,125]. These sleep disturbances not only diminish the quality of rest but can also impair cognitive performance the following day [122], creating a cycle where individuals may rely on further caffeine consumption to counteract sleep-related cognitive deficits. This cycle can lead to a dependency on caffeine and exacerbate the negative effects on both sleep and cognitive health in the long term.

In summary, energy drinks, due mainly to their caffeine content, can enhance lower cognitive functions and serve as a useful tool for maintaining alertness. However, their impact on sleep must be carefully managed. Excessive or poorly timed caffeine consumption can disrupt sleep patterns, leading to impaired cognitive performance. To maximize the cognitive benefits of energy drinks while minimizing their drawbacks, it is important to consider both the dosage and timing of their intake.

## 12. Should Pregnant Women Avoid Energy Drinks?

Pregnancy is a critical time of life for both the mother and the fetus. Nutritional needs change during pregnancy. Specific guidelines for pregnancy are created based on the health and safety of both mom and fetus. This includes restrictions on certain medications, foods, supplements, and caffeine. The American College of Obstetricians and Gynecologists (ACOG) recommends consuming <200 mg/day of caffeine during pregnancy [126], half of the current FDA recommendation (<400 mg/day) for healthy individuals. Caffeine intake during pregnancy is associated with acute changes in heart rate (HR), low birth weight, miscarriage, and birth defects [127,128,129]. The half-life of caffeine during pregnancy is longer and can range from 9 to 11 h during the third trimester [127,128]. Increased fetal HR, breathing, and a 2.3-fold increase in epinephrine levels were observed in fetuses after pregnant women consumed caffeine compared to decaffeinated coffee during the third trimester [130]. Multiple studies report similar increases in fetal HR following caffeine consumption [131,132].Caffeine increases the risk of uterine contractions, which may result in miscarriage [133]. Two meta-analyses report an increased risk for spontaneous abortion/miscarriages in pregnant women who consume more than 100 mg of caffeine [134,135].

From a caffeine perspective, EDs are presumably safe, assuming total daily intake stays below the ACOG recommendation. The average ED contains 80–150 mg of caffeine per 8 ounces [136]. However, it is essential to note that most EDs contain other ingredients that may be contraindicated for pregnancy. Assessing the direct effects of EDs on adverse pregnancy outcomes (APOs) is challenging. Numerous studies on caffeine exist, but few specifically involve EDs. Ding et al. [137] used a food frequency questionnaire to assess ED intake before and during pregnancy. Subjects were recruited from ongoing prospective studies (the Nurses’ Health Study 3 (NHS3) and the Growing Up Today Study (GUTS)). A correlation between pre-pregnancy ED consumption and gestational hypertension was reported. No correlations in other outcomes (pregnancy loss, preterm birth, gestational diabetes, preeclampsia) and ED consumption pre- and during pregnancy were reported. It is important to note that these findings were based on self-reported intakes and that the correlation in gestational hypertension was only reported in the NHS3 population.

Although one could take the position that EDs with only caffeine may be safe during pregnancy, it is better to err on the side of caution. Numerous caffeine-containing foods and beverages (chocolate, soda, tea, coffee, etc.) are consumed during pregnancy [127]. The addition of an ED is likely to exceed the current daily recommendation. The ACOG states that moderate caffeine consumption (<200 mg/day) is not a “major” factor in miscarriage or preterm birth. However, evidence suggests that caffeine intake may negatively impact the fetus [126,128,129,133,134].

In summary, based on the current caffeine guidelines and available literature, energy drinks are not recommended during pregnancy as even the small risk of an adverse event could result in irreversible consequences.

## 13. Do Energy Drinks Adversely Affect Cardiovascular Function?

Numerous studies have reported that caffeine consumption leads to a temporary increase in blood pressure (BP) and heart rate (HR) [14]. These changes are somewhat dose-dependent, with higher caffeine content correlating with more pronounced effects [138]. Caffeine has also been linked to an increase in myocardial oxygen demand, which may precipitate cardiac events in those with pre-existing cardiovascular conditions [139]. While moderate caffeine intake (100–400 mg daily) has been associated with either a neutral or protective effect on cardiovascular health in some studies, the acute consumption of higher doses, as found in EDs, may pose risks, particularly for individuals with underlying cardiovascular conditions [140]. Furthermore, certain individuals, especially those with genetic predispositions or pre-existing cardiovascular disease, might be more susceptible to caffeine-induced hypertension and other adverse cardiovascular effects.

Much of the concern over potential negative cardiovascular system impacts with EDs stems from reports of adverse events, which were evaluated by a systematic review and meta-analysis [141]. The authors found that, among adverse events reported by studies, the most common cardiorespiratory events in pediatric populations were chest pain (19.6%), palpitations (17.5%), dyspnea (17.1%), and tachycardia (12.5%) [141]. Of the events reported for the adult population, tachycardia (56.6%), palpitations (20.7%), and chest pain (4.9%) were the most reported cardiorespiratory complaints [141]. Of note, co-ingestion with alcohol was reported to significantly increase the stimulant effects of Eds [141].

In addition to the documented negative subjective cardiovascular events, a recent meta-analysis by Gualberto et al. [142] found evidence for objectively measured hemodynamic responses that may warrant some concern, particularly in populations with or at risk for hypertension or other cardiovascular disease. Analyses revealed that systolic blood pressure (SBP) significantly increased by up to 4.71 mmHg and diastolic blood pressure (DBP) by 4.51 mmHg within two hours of ED ingestion [142]. Sub-analyses indicated that this effect occurred whether the drink was classified as “low caffeine” (<196 mg) or “high caffeine” (>196 mg). An increase in cardiac output of up to 0.43 L/min was seen within 60 min of drink ingestion [142]. However, no effects on QT/QTc interval or endothelial function were apparent.

This latter finding is consistent with those of Elitok et al. [143] who found no impact on left ventricular repolarization (as assessed by Tp-e interval and Tp-e/QTc ratio) despite increases in SBP, DBP, and HR after acute ingestion of Red Bull ED. Contrary to this, Fletcher et al. [144] did find a significant difference in baseline-adjusted QTc 2 h after ingesting an ED with higher caffeine content (320 mg compared to 114 mg in the study by Elitok et al.). Importantly, it does not appear that this effect was due entirely to the caffeine content as this response, along with the peripheral SBP response, was not seen in a caffeine-only group matched for total caffeine content. There were no differences in peripheral DBP between the groups, though. It may be that the cardiovascular adverse effects may be due to the combined effects or interactions of the ingredients in EDs, potentially creating unique influences.

Taurine, another common ingredient in EDs, is an amino acid that plays a role in various physiological processes, including antioxidant and anti-inflammatory properties, blood pressure reduction, and the modulation of calcium signaling in the heart [145]. While taurine has been studied for its potential cardioprotective effects, particularly in patients with heart failure or other cardiovascular disease [145,146,147], its impact when consumed in the quantities and combinations present in EDs remains a topic of debate. There is also little to no evidence that taurine has positive cardiovascular effects in otherwise healthy individuals. Further, those who would seem to benefit the most from taurine supplementation’s potential cardiac benefit are those for whom ED consumption is most contraindicated.

EDs often contain B vitamins, particularly B6, B12, and folic acid, which are involved in energy metabolism [148]. While these vitamins are generally considered safe and even beneficial in reducing homocysteine levels [148]—a risk factor for cardiovascular disease—there is limited evidence to suggest that they have any significant cardiovascular effects when consumed in the context of ED. According to a systematic review by Miao et al. [149] higher intakes of B vitamins have been associated with a lower risk of cardiovascular disease in the general population. However, the beneficial effects of B vitamins may be overshadowed by the potential adverse effects of other ingredients in EDs, particularly when consumed in large quantities.

Guarana and ginseng are herbal ingredients commonly added to EDs for their stimulant properties [17]. Guarana contains caffeine, which contributes to the overall caffeine content of the beverage, while ginseng is believed to enhance physical and mental performance. Studies on guarana [150], have shown that it can lead to significant increases in SBP, similar to the effects of caffeine. Ginseng, on the other hand, has shown mixed results in clinical trials. Shah et al. [151] reported no significant differences in HR or BP following ginseng consumption compared to placebo, suggesting that its cardiovascular effects might be minimal when consumed alone. However, when combined with other stimulants in EDs, the overall impact on cardiovascular health could be more pronounced.

In summary, the scientific evidence suggests that energy drinks, primarily due to their high caffeine content coupled with ingredient interaction, can cause significant acute cardiovascular effects, including increases in blood pressure and heart rate. While ingredients like taurine and B vitamins may have some cardioprotective properties, their presence in energy drinks does not appear to mitigate the potential risks posed by high doses of caffeine and other stimulants like guarana.

## 14. Does Consuming Energy Drinks Cause Brain Damage?

The primary active compound in most EDs is caffeine, the effects of which have been widely discussed in the literature. According to Alsunni et al. [136] doses exceeding 200 mg of caffeine can lead to symptoms such as anxiety, insomnia, gastrointestinal disturbances, and restlessness. Prolonged high intake is linked to headaches and, in severe cases, psychiatric disorders induced by caffeine. In a preclinical study conducted by Sayed et al. [152], Red Bull administered at 10 mg/kg/day (equivalent to 7.5 mL per day for rats) was shown to induce structural injuries in the rat cerebral cortex, likely through oxidative stress. Translating this dose to human equivalents, a 100 kg individual would need to consume approximately 750 mL (or three small cans of Red Bull) to match the levels used in this study. It should be noted that studies showing a negative effect of EDs on brain health are typically done in animal models [153,154,155,156,157].

Notable case studies have highlighted the potential dangers of EDs. Several side effects, including elevated heart rate, increased blood pressure, anxiety, sleep disturbances, heart palpitations, and even seizures, have been documented in relation to excessive consumption of ED [158,159,160,161,162,163,164,165,166]. Specterman et al. [167] examined the effects of an ED containing glucose and caffeine on brain function using motor-evoked potentials (MEPs) derived from transcranial magnetic stimulation (TMS). Ten volunteers were tested, and the results indicated Lucozade (an ED with 68 g glucose, 46 mg caffeine) stem from the combined impact of glucose and caffeine on the brain via corticospinal excitability. It should, however, be pointed out that this dose of caffeine is much less than a typical serving of caffeinated coffee, and the amount of carbohydrate consumed is often much higher among exercising individuals [168,169].

The long-term effects of ED consumption on brain health remain an area of active investigation. While no conclusive evidence directly links EDs to structural brain damage, there is growing concern about the cognitive impacts of frequent, high-dose consumption. For instance, a cross-sectional survey of 8210 students in Atlantic Canada revealed that regular ED consumers reported higher levels of stress, anxiety, and depression compared to non-consumers [170]. If these mental health issues are left unchecked, they could exacerbate cognitive impairments over time, potentially leading to long-term dysfunction. A review by Richard and Smith [165] found that while the acute effects of ED consumption on mood are often positive, chronic consumption has been linked to increased levels of stress, anxiety, and depression. This aligns with broader concerns regarding the long-term mental health implications of regular ED use.

Conversely, if EDs do indeed pose a health risk due primarily to their caffeine content, one might expect that this would also occur with chronic coffee consumption, which typically contains more caffeine than an ED. However, this tends not to be the case. It is suggested that coffee consumption may reduce neuroinflammation, which is closely associated with the development of neurodegenerative disorders such as Alzheimer’s disease (AD), Parkinson’s disease (PD), multiple sclerosis (MS), amyotrophic lateral sclerosis (ALS), and Huntington’s disease (HD) [171].

In summary, while existing peer-reviewed studies highlight potential cognitive and behavioral effects associated with energy drinks, no evidence conclusively demonstrates that these beverages cause brain damage in humans. However, further research on brain functional changes and specific biomarkers such as glial fibrillary acidic protein (GFAP), neuron-specific enolase, and neurofilament light chain is warranted to clarify the neurological risks associated with energy drink consumption.

## 15. What Are Other Safety Considerations Regarding Energy Drinks?

ED consumption among individuals under 18 years has raised questions regarding their safety. Research indicates that EDs can be harmful to adolescents due to their stage of physiological development and increased sensitivity to stimulants [172,173,174,175,176,177,178,179]. Scientists and healthcare professionals took over a decade to question targeted marketing to children and adolescents, and since then, scrutiny has continued to increase.

Teenagers are still undergoing critical stages of cardiovascular and nervous system development. Caffeine affects the central nervous system by blocking adenosine receptors, leading to increased alertness and a temporary boost in energy [180]. Adolescents metabolize caffeine more slowly than adults, making them more sensitive to its effects. This heightened sensitivity can lead to cardiovascular changes, including increased heart rate and elevated blood pressure [141,181].

Adequate sleep impacts physical development, mood regulation, and cognitive function. EDs with a high caffeine content can significantly disrupt sleep patterns [176,182,183,184]. Chronic sleep deprivation has been linked to diminished mental performance, impaired memory, and reduced attention span independent of age 15, all of which can adversely affect academic performance and daily functioning.

In adolescents and young adults (aged 13–25), ED consumption resulted in frequent adverse cardiovascular and hemodynamic effects [185], behavioral issues, and arterial stiffness in those aged 10–18 [181] and physiological effects such as palpitations, chest pains, and sleep disturbances [160]. Adverse outcomes such as kidney failure, respiratory disorders, seizures, psychotic conditions, tachycardia, cardiac dysrhythmias, confusion, and even death were more common with caffeine levels above 200 mg and up to 1622 mg/day [175]. There was an increase in blood sugar levels, systolic and diastolic blood pressure, sleep disturbances, and pain tolerance in college students [186,187,188]. Young individuals (aged 22.3 ± 3.0) resulted in a prolonged QTc interval after 4 h of ED consumption, increasing the risk for arrhythmias [151,189]. There does not appear to be any therapeutic benefit of EDs for adolescents and young adults due to the unknown pharmacology of their ingredients [175].

Regular consumption of EDs can lead to caffeine dependence, a condition marked by the need to consume caffeine to avoid withdrawal symptoms [190]. Developing adolescent brain chemistry and the tendency for habitual behaviors to form during teenage years are particularly susceptible to addiction [172,179,180,191]. Dependence on caffeine can lead to withdrawal symptoms such as headaches, irritability, and fatigue, creating a cycle where individuals increasingly rely on EDs to function. EDs can also have significant impacts on mental health. The high caffeine content in susceptible individuals can exacerbate anxiety disorders and contribute to mood swings, as well as other psychological issues [165,192,193,194]. Furthermore, the combination of caffeine with other stimulants often found in EDs, such as taurine and guarana, may amplify these effects. Some studies suggest a correlation between ED consumption and increased risk of depression and behavioral problems in adolescents [176,180,182].

Young adults 26–40 years who consumed a mixed vitamin ED prolonged the QT interval [144,195], increased systolic blood pressure (SBP) after hours of consumption [144,196,197], and decreased postexposure insulin sensitivity [198], increasing the risk for fatal arrhythmias and adverse cardiovascular outcomes. However, ED consumption in males 25 and older increased fat oxidation, HR, and SBP during recovery without changes to ECG measures [199]. Additionally, there was an increase in the catecholamine pathway in individuals 18 and older who consumed EDs which could predispose to increased cardiovascular risk outcomes [200].

In older adults (>35 years), ED consumption is linked to severe symptoms such as cardiovascular, renal [201], and metabolic syndrome, leading to hospitalization and mortality compared to young adults [136,165,200,202]. The increased prevalence of ED consumption in older adults necessitates further research to determine if the observed effects are unique to young adults or also present in older populations [203]. The physiological effects of EDs cannot be easily attributed to single components or ingredient mixtures. Future research is needed to understand the long-term physiological effects on different age groups and to develop effective health preventive strategies.

In summary, individuals must be aware of the potential risks of energy drinks. The long-term effects of these immediate cardiovascular responses following the consumption of multiple energy drinks over an extended period (months or years) remain unknown. Additionally, most of the research on this subject has concentrated on young, healthy adults, leaving the impact on those with comorbidities, especially cardiovascular or metabolic diseases, unclear.

## 16. Is There Any Evidence to Suggest That Energy Drinks Are More Effective than an Identical Serving of Caffeine Alone?

The data demonstrates a difference in how the body responds to vascular tone between energy drinks and caffeine. Energy drinks appear to impact vascular tone in a potentially negative way, while caffeine does not have the same effect [204]. A similar study found no difference between the energy drink and caffeine on cardiac function as measured by blood pressure and the QTc interval [205].

Aside from limited evidence supporting glucose and guaraná extract, there is a lack of evidence to back claims that ingredients in EDs, other than caffeine, enhance physical or cognitive performance [3]. Fletcher et al. conducted a randomized, double-blind, controlled crossover study with 18 young, healthy participants. Each participant consumed either 946 mL (32 ounces) of ED or a caffeinated control drink, both containing 320 mg of caffeine, with a 6-day washout period between tests. An electrocardiogram (ECG), peripheral blood pressure (BP), and central BP measurements were taken at baseline and 1, 2, 4, 6, and 24 h after consumption. Changes from baseline were compared between the two groups [144]. The corrected QT interval increased significantly more in the energy drink group than in the caffeine group at 2 h. No significant QTc differences were observed at other time points. Both the ED and caffeine groups experienced similar initial systolic BP increases, but at 6 h, systolic BP was significantly higher in the energy drink group. There were no significant differences in heart rate, diastolic BP, central systolic BP, or central diastolic BP between the two groups at any time [144].

Work by Pereira et al. directly compared an ED with a positive control that contained an equal serving of caffeine (200 mg) [64]. In a randomized, counterbalanced, crossover study, participants first completed assessments of mood (Profile of Mood States; POMS), sustained attention (Psychomotor Vigilance Test; PVT), handgrip strength (HG), and 1-min maximum push-up performance (PU). They then consumed an energy drink (ED) or a caffeine-only beverage (CAF) containing 200 mg of caffeine in a randomized sequence. After 45 min, the assessments were repeated. Following a 1-week washout, participants returned to consume the alternate drink and repeated the protocol. Results showed that while the ED group improved reaction time on the PVT, the difference in delta scores between ED and CAF was not statistically significant. Additionally, no significant differences were observed between ED and CAF groups for mood, handgrip strength, or push-up performance. These findings indicate that the added ingredients in the ED may not provide substantial benefits over caffeine alone [64].

In summary, there does appear to be a difference between energy drinks versus an equal serving of caffeine regarding the cardiovascular response. Moreover, data are scarce regarding the ergogenic effects of energy drinks compared to an equal serving of caffeine. Nonetheless, one can reasonably posit that caffeine is the primary driver of any effects regarding energy drinks.

## 17. If Caffeine Is the Main Active Ingredient in Energy Drinks and Coffee, Why Is There a Discrepancy in the Adverse Events Reported for Each?

The answer to this question depends on several things. Science is reductionist in nature and requires operational definitions. This is not easily done for food products, which are often complex matrices of many compounds. Although the ingredient of interest in EDs and “regular” coffee is commonly considered to be caffeine, one can attempt to define each of these beverages further. First, the term energy drink does not connote just one thing. There are myriad formulae among competing products, containing various ingredients and doses: Caffeine, sugars, amino acids, metabolites, vitamins, botanicals, and even medicinal mushrooms [17,206,207]. Similarly, coffee is not just one thing: bean type, roast, grind, brew method, and additives like sugar and milk affect what is in the cup [208]. Indeed, even the term “cup” is debatable (see below). Of the hundreds of compounds in coffee other than caffeine, research has focused on polyphenols such as caffeic and chlorogenic acids, diterpenes such as cafestol and kahweol, the alkaloid trigonelline—which is structurally related to nicotinic acid—and Maillard reaction (browning) products called melanoidins [208,209].

Energy drinks, being synthesized and dosed by various companies, are generally defined as containing over 150 mg of caffeine per liter [210]. The breadth of ED products makes assessing caffeine content difficult, but McLellan and Lieberman (2012) suggested it typically be 40–235 mg per 235 mL serving [3]. In comparison, “natural” brewed arabica coffee generally contains ~95 mg caffeine per 8 oz. (237 g) cup [211]. The energy-drink-to-coffee comparison regarding caffeine dose can be difficult to apply to daily life, however, because coffees vary greatly, too. As described by McCusker and colleagues: “Another notable find is the wide range of caffeine concentrations (259–564 mg/dose) in the same coffee beverage obtained from the same outlet on six consecutive days” [212].

It is important to understand that the scientific literature on energy drinks has tended to include negativity or concern over the years [141,206] whilst publications on coffee ingredients have largely moved in the opposite direction, focusing on benefits [208,213,214]. This has been influenced by positive findings on the antioxidant, neuroprotective, potentially muscle-preserving, and potentially anti-diabetic effects of the complex matrix that is “coffee” [208].

According to Jagim et al., potential adverse events from EDs include various cardiovascular adverse effects, insomnia, stress, jitteriness/restlessness, depression, and gastrointestinal upset [17]. Each of these effects is known to be partly attributable to caffeine, directly or indirectly, as contributed by various botanical ingredients such as guarana and kola nut. This is not to say that the complex matrix of ingredients in food products does not play a role; interactions depend on the formulation. For the consumer, medical history, genetic predisposition, lifestyle, and possibly sex affect responses, as do acute versus chronic considerations [2,17,208]. Specific to EDs, according to Nadeem et al. [141], elevated heart rate (tachycardia) was the most frequently reported cardiorespiratory event (26.2%), followed by heart palpitations (20.0%). Neural adverse events included headaches as the most frequent event (18.4%), followed by dizziness (12.3%), and tremors (11.4%). Related physiological events include sleep disturbances (34.5%), and restlessness/shaking hands (25.1%) [141]. Of these, heart rate and rhythm abnormalities (arrythmias/palpitations) may be the most physiologically serious for consumers.

Observational data reported by Chieng and colleagues from a United Kingdom database showed that ground and instant coffee consumption was associated with a significant *reduction* in arrhythmias at 1–5 cups/day [213]. According to Surma, et al., there are pro- and anti-arrhythmogenic effects of coffee, with the antiarrhythmic properties of coffee potentially due in part to its influence on the composition of the gut microbiota [215]. These authors also summarized the relationship between coffee consumption and the risk of arrhythmias across several meta-analyzes; they concluded no/reduced effect of coffee consumption on the risk of new-onset atrial fibrillation and no evidence that coffee consumption influenced the rate of ventricular premature beats [215]. According to the International Society of Sports Nutrition (ISSN) position stand on coffee, the beverage has mild effects on heart rate and blood pressure in healthy active persons but can precipitate increased anxiety, jitters and sleep disturbances depending on individual differences, dose, and timing [208]. However, these authors also note paradoxical effects: “Coffee contains dopaminergic components beyond caffeine… coffee’s polyphenolic components appear to be neuroprotective and to beneficially influence mood, including reduced anxiety scores and depression”. Thus, although coffee’s stimulant nature can induce certain adverse events in susceptible people, similar to those attributed to EDs (acute anxiousness, jitters, sleep disturbances), coffee’s bioactives may provide protection and/or other benefits against cardiovascular and neural symptoms. This hypothesis may be reflected in data sets such as the one published by Jagim et al. that suggested far fewer reported adverse events for coffee/tea/soda versus energy products [216]. This was true of both total and severe adverse events. Of course, such findings could also be ascribed to a lower caffeine dose in the combined coffee/tea/soda category. Again, the dose and ingredients matter.

In summary, large variability among energy drinks and coffees prevents a definitive conclusion on the nature of the adverse events reported for energy drinks. On the other hand, research on coffee has tended to be more favorable. Consumers sensitive to stimulants should exercise caution with either type of beverage. Assuming caffeine is the commonality among (the wide range of) energy drinks and coffees, the caffeine dose is likely to be a key driver of adverse events in susceptible individuals.

## 18. Conclusions

Based on our evidence-based scientific evaluation of the literature, we conclude that:

- Caffeine, sugar, taurine, and B vitamins are several of the more common ingredients found in energy drinks. Caffeine is the single most important ingredient.
- It is reasonable to conclude that EDs do not impact body composition.
- The available evidence suggests that energy drinks may provide modest benefits to endurance performance, particularly for longer-duration activities.
- The use of strategic caffeinated EDs at a relative dose >3 mg·kg^−1^ demonstrates evidence of enhanced athletic speed in individual and team sports.
- There is evidence that energy drinks containing caffeine can improve visual and auditory reaction time, although results are mixed.
- There is evidence that consuming commercially available energy drinks can improve upper and lower body muscle endurance and power in young healthy adults. However, there is no evidence that energy drinks can increase lean tissue mass in humans.
- Several studies have evaluated the effects of energy drinks on cognitive performance, showing improved alertness, concentration, memory, and attention.
- Various commercially available energy drinks can acutely increase resting energy expenditure up to three hours after ingestion.
- There is limited evidence to suggest the presence of sex differences in response to energy drinks.
- Excessive or poorly timed caffeine consumption can disrupt sleep patterns and thus impair cognitive performance.
- Energy drinks are not recommended during pregnancy as even the small risk of an adverse event could result in irreversible consequences to the child.
- The evidence suggests that energy drinks, primarily due to their high caffeine content coupled with ingredient interaction, can cause significant acute cardiovascular effects, including increases in blood pressure and heart rate.
- There is no evidence that these beverages cause brain damage in humans.
- Individuals must be aware of the potential risks of energy drinks. The long-term effects of these immediate cardiovascular responses following the consumption of multiple energy drinks over an extended period (months or years) remain unknown.
- Data are scarce regarding the ergogenic effects of energy drinks compared to an equal serving of caffeine. However, it is likely that caffeine alone provides the ergogenic effect.
- In summary, large variability among energy drinks and coffees prevents a definitive conclusion on the nature of the adverse events reported for energy drinks. Assuming caffeine is the commonality among energy drinks and coffees, the caffeine dose is likely to be a key driver of adverse events in susceptible individuals.

## Figures and Tables

**Table 1:** Prevalence and quantities of ingredients included in the bestselling energy drinks and energy shots (*n* = 75) per serving size. Reproduced from Jagim et al., 2022 [16], which is licensed under an open-access Creative Commons CC BY 4.0 license.

Ingredient	Overall Prevalence (%)	Prevalence in Undisclosed Quantity (%)	Prevalence in Listed Quantity (%)	Mean ± SD Listed Quantity	Range
Caffeine (mg)	100	0	100	174 ± 81	45; 400
Vitamin B6 (% DV)	72.0	0	72	367 ± 648	17.7; 2353
Sodium (mg)	70.7	0	70.7	120 ± 118	2; 530
Niacin (% DV)	66.7	0	66.7	121 ± 70	18.8; 250
Vitamin B12 (% DV)	66.7	0	66.7	5245 ± 10,475	18.8; 41,666.7
Sugars (g)	45.3	0	45.3	19.9 ± 18.2	1; 63
Vitamin B5 (% DV)	37.3	0	37.3	114 ± 77	20; 400
Taurine	37.3	37.3	0	N/A	N/A
Potassium (mg)	34.7	0	34.7	148 ± 197	13; 830
Ginseng	30.7	30.7	0	N/A	N/A
Guarana	25.3	25.3	0	N/A	N/A
Vitamin C (% DV)	22.7	0	22.7	59.8 ± 48.7	3; 190
Tyrosine	22.7	22.7	0	N/A	N/A
Calcium (mg)	17.3	0	17.3	128 ± 175	5; 520
L-Theanine	17.3	17.3	0	N/A	N/A
Carnitine	16.0	16.0	0	N/A	N/A
Magnesium (mg)	12.0	0	12.0	25.4 ± 23.4	3.5; 74
Vitamin B2 (% DV)	8.0	0	8.0	133 ± 81	40; 260
Vitamin A (% DV)	6.7	0	6.7	78.6 ± 86.8	10; 220
Folate (mcg)	6.7	0	6.7	258 ± 194	40; 400
Vitamin D (% DV)	2.7	0	2.7	35.0 ± 21.2	20; 50
Choline (mg)	2.7	0	2.7	267 ± 330	33; 500
Vitamin B1 (% DV)	1.3	0	1.3	25.0	N/A

## Data Availability

Not applicable.

## References

[1] Astorino T.A., Roberson D.W. (2010). Efficacy of Acute Caffeine Ingestion for Short-term High-Intensity Exercise Performance: A Systematic Review. J. Strength Cond. Res..

[2] Guest N.S., VanDusseldorp T.A., Nelson M.T., Grgic J., Schoenfeld B.J., Jenkins N.D.M., Arent S.M., Antonio J., Stout J.R., Trexler E.T. (2021). International society of sports nutrition position stand: Caffeine and exercise performance. J. Int. Soc. Sports Nutr..

[3] McLellan T.M., Lieberman H.R. (2012). Do energy drinks contain active components other than caffeine?. Nutr. Rev..

[4] Grgic J., Grgic I., Pickering C., Schoenfeld B.J., Bishop D.J., Pedisic Z. (2020). Wake up and smell the coffee: Caffeine supplementation and exercise performance-an umbrella review of 21 published meta-analyses. Br. J. Sports Med..

[5] Polito M.D., Souza D.B., Casonatto J., Farinatti P. (2016). Acute effect of caffeine consumption on isotonic muscular strength and endurance: A systematic review and meta-analysis. Sci. Sports.

[6] Crowe M.J., Leicht A.S., Spinks W.L. (2006). Physiological and cognitive responses to caffeine during repeated, high-intensity exercise. Int. J. Sport Nutr. Exerc. Metab..

[7] Graham T.E. (2001). Caffeine and exercise: Metabolism, endurance and performance. Sports Med..

[8] Lopes J.M., Aubier M., Jardim J., Aranda J.V., Macklem P.T. (1983). Effect of caffeine on skeletal muscle function before and after fatigue. J. Appl. Physiol. Respir. Environ. Exerc. Physiol..

[9] Boswell-Smith V., Spina D., Page C.P. (2006). Phosphodiesterase inhibitors. Br. J. Pharmacol..

[10] Jackman M., Wendling P., Friars D., Graham T.E. (1996). Metabolic catecholamine, and endurance responses to caffeine during intense exercise. J. Appl. Physiol. (1985).

[11] Kurtz J.A., VanDusseldorp T.A., Doyle J.A., Otis J.S. (2021). Taurine in sports and exercise. J. Int. Soc. Sports Nutr..

[12] Henselmans M., Bjørnsen T., Hedderman R., Vårvik F.T. (2022). The Effect of Carbohydrate Intake on Strength and Resistance Training Performance: A Systematic Review. Nutrients.

[13] Forbes S.C., Candow D.G., Little J.P., Magnus C., Chilibeck P.D. (2007). Effect of Red Bull Energy Drink on Repeated Wingate Cycle Performance and Bench-Press Muscle Endurance. Int. J. Sport Nutr. Exerc. Metab..

[14] Antonio J., Newmire D.E., Stout J.R., Antonio B., Gibbons M., Lowery L.M., Harper J., Willoughby D., Evans C., Anderson D. (2024). Common questions and misconceptions about caffeine supplementation: What does the scientific evidence really show?. J. Int. Soc. Sports Nutr..

[15] Antonio J., Evans C., Ferrando A.A., Stout J.R., Antonio B., Cintineo H.P., Harty P., Arent S.M., Candow D.G., Forbes S.C. (2024). Common questions and misconceptions about protein supplementation: What does the scientific evidence really show?. J. Int. Soc. Sports Nutr..

[16] Jagim A.R., Harty P.S., Barakat A.R., Erickson J.L., Carvalho V., Khurelbaatar C., Camic C.L., Kerksick C.M. (2022). Prevalence and Amounts of Common Ingredients Found in Energy Drinks and Shots. Nutrients.

[17] Jagim A.R., Harty P.S., Tinsley G.M., Kerksick C.M., Gonzalez A.M., Kreider R.B., Arent S.M., Jager R., Smith-Ryan A.E., Stout J.R. (2023). International society of sports nutrition position stand: Energy drinks and energy shots. J. Int. Soc. Sports Nutr..

[18] Caine J.J., Geracioti T.D. (2016). Taurine, energy drinks, and neuroendocrine effects. Cleve Clin. J. Med..

[19] Kontny E., Szczepanska K., Kowalczewski J., Kurowska M., Janicka I., Marcinkiewicz J., Maslinski W. (2000). The mechanism of taurine chloramine inhibition of cytokine (interleukin-6, interleukin-8) production by rheumatoid arthritis fibroblast-like synoviocytes. Arthritis Rheum..

[20] Kammerer M., Jaramillo J.A., Garcia A., Calderon J.C., Valbuena L.H. (2014). Effects of energy drink major bioactive compounds on the performance of young adults in fitness and cognitive tests: A randomized controlled trial. J. Int. Soc. Sports Nutr..

[21] Jagim A.R., Harty P.S., Camic C.L. (2019). Common Ingredient Profiles of Multi-Ingredient Pre-Workout Supplements. Nutrients.

[22] Westerterp-Plantenga M.S. (2010). Green tea catechins, caffeine and body-weight regulation. Physiol. Behav..

[23] Dalbo V.J., Roberts M.D., Stout J.R., Kerksick C.M. (2008). Acute effects of ingesting a commercial thermogenic drink on changes in energy expenditure and markers of lipolysis. J. Int. Soc. Sports Nutr..

[24] Dalbo V.J., Roberts M.D., Stout J.R., Kerksick C.M. (2010). Effect of gender on the metabolic impact of a commercially available thermogenic drink. J. Strength Cond. Res..

[25] Harty P.S., Stratton M.T., Escalante G., Rodriguez C., Dellinger J.R., Williams A.D., White S.J., Smith R.W., Johnson B.A., Sanders M.B. (2020). Effects of Bang(R) Keto Coffee Energy Drink on Metabolism and Exercise Performance in Resistance-Trained Adults: A Randomized, Double-blind, Placebo-controlled, Crossover Study. J. Int. Soc. Sports Nutr..

[26] Rashti S.L., Ratamess N.A., Kang J., Faigenbaum A.D., Chilakos A., Hoffman J.R. (2009). Thermogenic effect of meltdown RTD energy drink in young healthy women: A double blind, cross-over design study. Lipids Health Dis..

[27] Roberts M.D., Dalbo V.J., Hassell S.E., Stout J.R., Kerksick C.M. (2008). Efficacy and safety of a popular thermogenic drink after 28 days of ingestion. J. Int. Soc. Sports Nutr..

[28] Lockwood C.M. (2010). Effect of Whey Protein on Physiological Response to Chronic Resistance Exercise in Trained Men: A Double-Blind, Placebo Controlled, Randomized Trial.

[29] Smith A.E., Lockwood C.M., Moon J.R., Kendall K.L., Fukuda D.H., Tobkin S.E., Cramer J.T., Stout J.R. (2010). Physiological effects of caffeine, epigallocatechin-3-gallate, and exercise in overweight and obese women. Appl. Physiol. Nutr. Metab..

[30] Siedler M.R., Rodriguez C., White S.J., Tinoco E., DeHaven B., Brojanac A., LaValle C., Rasco J., Taylor L.W., Tinsley G.M. (2023). Chronic Thermogenic Dietary Supplement Consumption: Effects on Body Composition, Anthropometrics, and Metabolism. Nutrients.

[31] Ivy J.L., Kammer L., Ding Z., Wang B., Bernard J.R., Liao Y.H., Hwang J. (2009). Improved cycling time-trial performance after ingestion of a caffeine energy drink. Int. J. Sport Nutr. Exerc. Metab..

[32] Geiß K.R., Jester I., Falke W., Hamm M., Waag K.L. (1994). The effect of a taurine-containing drink on performance in 10 endurance-athletes. Amino Acids.

[33] Del Coso J., Muñoz-Fernández V.E., Muñoz G., Fernández-Elías V.E., Ortega J.F., Hamouti N., Barbero J.C., Muñoz-Guerra J. (2012). Effects of a caffeine-containing energy drink on simulated soccer performance. PLoS ONE.

[34] Prins P.J., Koutnik A.P., D’Agostino D.P., Rogers C.Q., Seibert J.F., Breckenridge J.A., Jackson D.S., Ryan E.J., Buxton J.D., Ault D.L. (2020). Effects of an Exogenous Ketone Supplement on Five-Kilometer Running Performance. J. Hum. Kinet..

[35] Del Coso J., Ramírez J.A., Muñoz G., Portillo J., Gonzalez-Millán C., Muñoz V., Barbero-Álvarez J.C., Muñoz-Guerra J. (2013). Caffeine-containing energy drink improves physical performance of elite rugby players during a simulated match. Appl. Physiol. Nutr. Metab..

[36] Sanders G.J., Peveler W., Holmer B., Peacock C.A. (2015). The effect of three different energy drinks on oxygen consumption and perceived exertion during treadmill exercise. J. Int. Soc. Sports Nutr..

[37] Candow D.G., Kleisinger A.K., Grenier S., Dorsch K.D. (2009). Effect of sugar-free Red Bull energy drink on high-intensity run time-to-exhaustion in young adults. J. Strength Cond. Res..

[38] Talanian J.L., Spriet L.L. (2016). Low and moderate doses of caffeine late in exercise improve performance in trained cyclists. Appl. Physiol. Nutr. Metab..

[39] Alford C., Cox H., Wescott R. (2001). The effects of red bull energy drink on human performance and mood. Amino Acids.

[40] Womack C.J., Saunders M.J., Bechtel M.K., Bolton D.J., Martin M., Luden N.D., Dunham W., Hancock M. (2012). The influence of a CYP1A2 polymorphism on the ergogenic effects of caffeine. J. Int. Soc. Sports Nutr..

[41] Gonçalves L.S., Painelli V.S., Yamaguchi G., Oliveira L.F., Saunders B., da Silva R.P., Maciel E., Artioli G.G., Roschel H., Gualano B. (2017). Dispelling the myth that habitual caffeine consumption influences the performance response to acute caffeine supplementation. J. Appl. Physiol..

[42] Del Coso J., Portillo J., Salinero J.J., Lara B., Abian-Vicen J., Areces F. (2016). Caffeinated energy drinks improve high-speed running in elite field hockey players. Int. J. Sport Nutr. Exerc. Metab..

[43] Del Coso J., Portillo J., Muñoz G., Abián-Vicén J., Gonzalez-Millán C., Muñoz-Guerra J. (2013). Caffeine-containing energy drink improves sprint performance during an international rugby sevens competition. Amino Acids.

[44] Lara B., Gonzalez-Millán C., Salinero J.J., Abian-Vicen J., Areces F., Barbero-Alvarez J.C., Muñoz V., Portillo L.J., Gonzalez-Rave J.M., Del Coso J. (2014). Caffeine-containing energy drink improves physical performance in female soccer players. Amino Acids.

[45] Gallo-Salazar C., Areces F., Abián-Vicén J., Lara B., Salinero J.J., Gonzalez-Millán C., Portillo J., Muñoz V., Juarez D., Del Coso J. (2015). Enhancing physical performance in elite junior tennis players with a caffeinated energy drink. Int. J. Sports Physiol. Perform..

[46] Lara B., Ruiz-Vicente D., Areces F., Abián-Vicén J., Salinero J.J., Gonzalez-Millán C., Gallo-Salazar C., Del Coso J. (2015). Acute consumption of a caffeinated energy drink enhances aspects of performance in sprint swimmers. Br. J. Nutr..

[47] Astorino T.A., Matera A.J., Basinger J., Evans M., Schurman T., Marquez R. (2012). Effects of red bull energy drink on repeated sprint performance in women athletes. Amino Acids.

[48] Temple J.L., Ziegler A.M. (2011). Gender Differences in Subjective and Physiological Responses to Caffeine and the Role of Steroid Hormones. J. Caffeine Res..

[49] Davis J.M., Zhao Z., Stock H.S., Mehl K.A., Buggy J., Hand G.A. (2003). Central nervous system effects of caffeine and adenosine on fatigue. Am. J. Physiol. -Regul. Integr. Comp. Physiol..

[50] Alansare A.B., Hayman J., Lee J.-M., Seo M.-W., Yoo D., Jung H.C. (2021). The efficacy of a calamansi-containing energy drink on running performance and recovery in NCAA division I middle-distance runners: A preliminary study. Int. J. Environ. Res. Public Health.

[51] Prins P.J., Goss F.L., Nagle E.F., Beals K., Robertson R.J., Lovalekar M.T., Welton G.L. (2016). Energy drinks improve five-kilometer running performance in recreational endurance runners. J. Strength Cond. Res..

[52] Gwacham N., Wagner D.R. (2012). Acute effects of a caffeine-taurine energy drink on repeated sprint performance of American college football players. Int. J. Sport Nutr. Exerc. Metab..

[53] Karayigit R., Naderi A., Saunders B., Forbes S.C., Coso J.D., Berjisian E., Yildirim U.C., Suzuki K. (2021). Combined but not isolated ingestion of caffeine and taurine improves Wingate Sprint Performance in female team-sport athletes habituated to caffeine. Sports.

[54] Schubert M.M., Astorino T.A., Azevedo Jr J.L. (2013). The effects of caffeinated “energy shots” on time trial performance. Nutrients.

[55] Erdmann J., Wiciński M., Wódkiewicz E., Nowaczewska M., Słupski M., Otto S.W., Kubiak K., Huk-Wieliczuk E., Malinowski B. (2021). Effects of energy drink consumption on physical performance and potential danger of inordinate usage. Nutrients.

[56] Ali F., Rehman H., Babayan Z., Stapleton D., Joshi D.-D. (2015). Energy drinks and their adverse health effects: A systematic review of the current evidence. Postgrad. Med..

[57] Lazarus M., Shen H.-Y., Cherasse Y., Qu W.-M., Huang Z.-L., Bass C.E., Winsky-Sommerer R., Semba K., Fredholm B.B., Boison D. (2011). Arousal effect of caffeine depends on adenosine A2A receptors in the shell of the nucleus accumbens. J. Neurosci..

[58] Goel V., Manjunatha S., Pai K.M. (2014). Effect of red bull energy drink on auditory reaction time and maximal voluntary contraction. Indian. J. Physiol. Pharmacol..

[59] Concerto C., Infortuna C., Chusid E., Coira D., Babayev J., Metwaly R., Naenifard H., Aguglia E., Battaglia F. (2017). Caffeinated energy drink intake modulates motor circuits at rest, before and after a movement. Physiol. Behav..

[60] Howard M.A., Marczinski C.A. (2010). Acute effects of a glucose energy drink on behavioral control. Exp. Clin. Psychopharmacol..

[61] Antonio J., Kenyon M., Horn C., Jiannine L., Carson C., Ellerbroek A., Roberts J., Peacock C., Tartar J. (2019). The effects of an energy drink on psychomotor vigilance in trained individuals. J. Funct. Morphol. Kinesiol..

[62] Howden J., Remme H. (2022). The Effect of Energy Drink Consumption on Brake Reaction Time. Sch. Horiz. Univ. Minn. Morris Undergrad. J..

[63] Evans C., Mekhail V., Kaminski J., Peacock C., Tartar J., Santana J.C., Antonio J. (2021). The Effects of an Energy Drink on Measures of Cognition and Physical Performance. J. Exerc. Physiol. Online.

[64] Pereira F., Evans C., Rojas J., Curtis J., Andal A., Thakkar H., Rocanelli R., Rodriguez C.C., Santana J.C., Jiannine L. (2024). Beyond the Buzz: Do Energy Drinks Offer More Than Caffeine for Mental and Physical Tasks?. Int. J. Exerc. Sci..

[65] Moore T.M., Mortensen X.M., Ashby C.K., Harris A.M., Kump K.J., Laird D.W., Adams A.J., Bray J.K., Chen T., Thomson D.M. (2017). The effect of caffeine on skeletal muscle anabolic signaling and hypertrophy. Appl. Physiol. Nutr. Metab..

[66] Lockwood C.M., Moon J.R., Smith A.E., Tobkin S.E., Kendall K.L., Graef J.L., Cramer J.T., Stout J.R. (2010). Low-Calorie Energy Drink Improves Physiological Response to Exercise in Previously Sedentary Men: A Placebo-Controlled Efficacy and Safety Study. J. Strength Cond. Res..

[67] Astley C., Souza D.B., Polito M.D. (2018). Acute Specific Effects of Caffeine-containing Energy Drink on Different Physical Performances in Resistance-trained Men. Int. J. Exerc. Sci..

[68] Del Coso J., Salinero J.J., González-Millán C., Abián-Vicén J., Pérez-González B. (2012). Dose response effects of a caffeine-containing energy drink on muscle performance: A repeated measures design. J. Int. Soc. Sports Nutr..

[69] Mets M.A., Ketzer S., Blom C., van Gerven M.H., van Willigenburg G.M., Olivier B., Verster J.C. (2011). Positive effects of Red Bull^®^ Energy Drink on driving performance during prolonged driving. Psychopharmacology.

[70] Wesnes K.A., Brooker H., Watson A.W., Bal W., Okello E. (2017). Effects of the Red Bull energy drink on cognitive function and mood in healthy young volunteers. J. Psychopharmacol..

[71] Cavka A., Stupin M., Panduric A., Plazibat A., Cosic A., Rasic L., Debeljak Z., Martinovic G., Drenjancevic I. (2015). Adrenergic System Activation Mediates Changes in Cardiovascular and Psychomotoric Reactions in Young Individuals after Red Bull (©) Energy Drink Consumption. Int. J. Endocrinol..

[72] Thomas C.J., Rothschild J., Earnest C.P., Blaisdell A. (2019). The Effects of Energy Drink Consumption on Cognitive and Physical Performance in Elite League of Legends Players. Sports.

[73] Buckenmeyer P.J., Bauer J.A., Hokanson J.F., Hendrick J.L. (2015). Cognitive influence of a 5-h ENERGY^®^ shot: Are effects perceived or real?. Physiol. Behav..

[74] Bloomer R., Majaj R., Moran R., MacDonnchadh J. (2015). Comparison of 5-Hour ENERGY and Caffeine on Cognitive Performance and Subjective Feelings in Young Men and Women. J. Caffeine Res..

[75] Garcia-Alvarez A., Cunningham C.A., Mui B., Penn L., Spaulding E.M., Oakes J.M., Divers J., Dickinson S.L., Xu X., Cheskin L.J. (2020). A randomized, placebo-controlled crossover trial of a decaffeinated energy drink shows no significant acute effect on mental energy. Am. J. Clin. Nutr..

[76] Kennedy D.O., Wightman E.L. (2022). Mental Performance and Sport: Caffeine and Co-consumed Bioactive Ingredients. Sports Med..

[77] Scholey A.B., Kennedy D.O. (2004). Cognitive and physiological effects of an “energy drink”: An evaluation of the whole drink and of glucose, caffeine and herbal flavouring fractions. Psychopharmacology.

[78] Adan A., Serra-Grabulosa J.M. (2010). Effects of caffeine and glucose, alone and combined, on cognitive performance. Hum. Psychopharmacol..

[79] Giles G.E., Mahoney C.R., Brunyé T.T., Gardony A.L., Taylor H.A., Kanarek R.B. (2012). Differential cognitive effects of energy drink ingredients: Caffeine, taurine, and glucose. Pharmacol. Biochem. Behav..

[80] Smit H.J., Cotton J.R., Hughes S.C., Rogers P.J. (2004). Mood and cognitive performance effects of “energy” drink constituents: Caffeine, glucose and carbonation. Nutr. Neurosci..

[81] Rao A., Hu H., Nobre A.C. (2005). The effects of combined caffeine and glucose drinks on attention in the human brain. Nutr. Neurosci..

[82] Childs E. (2014). Influence of energy drink ingredients on mood and cognitive performance. Nutr. Rev..

[83] Bernard B.N., Louise L.C., Louise D. (2018). The Effects of Carbohydrates, in Isolation and Combined with Caffeine, on Cognitive Performance and Mood-Current Evidence and Future Directions. Nutrients.

[84] Seidl R., Peyrl A., Nicham R., Hauser E. (2000). A taurine and caffeine-containing drink stimulates cognitive performance and well-being. Amino Acids.

[85] Ozan M., Buzdagli Y., Eyipinar C.D., Baygutalp N.K., Yüce N., Oget F., Kan E., Baygutalp F. (2022). Does Single or Combined Caffeine and Taurine Supplementation Improve Athletic and Cognitive Performance without Affecting Fatigue Level in Elite Boxers? A Double-Blind, Placebo-Controlled Study. Nutrients.

[86] Owen G.N., Parnell H., De Bruin E.A., Rycroft J.A. (2008). The combined effects of L-theanine and caffeine on cognitive performance and mood. Nutr. Neurosci..

[87] Giesbrecht T., Rycroft J.A., Rowson M.J., De Bruin E.A. (2010). The combination of L-theanine and caffeine improves cognitive performance and increases subjective alertness. Nutr. Neurosci..

[88] Mendel R.W., Hofheins J.E. (2007). Metabolic responses to the acute ingestion of two commercially available carbonated beverages: A pilot study. J. Int. Soc. Sports Nutr..

[89] Rodriguez C., Stratton M.T., Harty P.S., Siedler M.R., Boykin J.R., Green J.J., Keith D.S., White S.J., DeHaven B., Brojanac A. (2023). Effects of a ready-to-drink thermogenic beverage on resting energy expenditure, hemodynamic function, and subjective outcomes. J. Int. Soc. Sports Nutr..

[90] Clark N.W., Wells A.J., Coker N.A., Goldstein E.R., Herring C.H., Starling-Smith T.M., Varanoske A.N., Panissa V.L., Stout J.R., Fukuda D.H. (2020). The acute effects of thermogenic fitness drink formulas containing 140 mg and 100 mg of caffeine on energy expenditure and fat metabolism at rest and during exercise. J. Int. Soc. Sports Nutr..

[91] Del Coso J., Perez-Lopez A., Abian-Vicen J., Salinero J.J., Lara B., Valades D. (2014). Enhancing physical performance in male volleyball players with a caffeine-containing energy drink. Int. J. Sports Physiol. Perform..

[92] Abian-Vicen J., Puente C., Salinero J.J., Gonzalez-Millan C., Areces F., Munoz G., Munoz-Guerra J., Del Coso J. (2014). A caffeinated energy drink improves jump performance in adolescent basketball players. Amino Acids.

[93] Eckerson J.M., Bull A.J., Baechle T.R., Fischer C.A., O’Brien D.C., Moore G.A., Yee J.C., Pulverenti T.S. (2013). Acute ingestion of sugar-free red bull energy drink has no effect on upper body strength and muscular endurance in resistance trained men. J. Strength Cond. Res..

[94] Fernandez-Campos C., Dengo A.L., Moncada-Jimenez J. (2015). Acute Consumption of an Energy Drink Does Not Improve Physical Performance of Female Volleyball Players. Int. J. Sport Nutr. Exerc. Metab..

[95] Kazemi F., Gaeini A.A., Kordi M.R., Rahnama N. (2009). The acute effects of two energy drinks on endurance performance in female athlete students. Sport Sci. Health.

[96] Jacobson B.H., Hester G.M., Palmer T.B., Williams K., Pope Z.K., Sellers J.H., Conchola E.C., Woolsey C., Estrada C. (2017). Effect of Energy Drink Consumption on Power and Velocity of Selected Sport Performance Activities. J. Strength Cond. Res..

[97] Nelson M.T., Biltz G.R., Dengel D.R. (2014). Cardiovascular and ride time-to-exhaustion effects of an energy drink. J. Int. Soc. Sports Nutr..

[98] Salinero J.J., Lara B., Abian-Vicen J., Gonzalez-Millán C., Areces F., Gallo-Salazar C., Ruiz-Vicente D., Del Coso J. (2014). The use of energy drinks in sport: Perceived ergogenicity and side effects in male and female athletes. Br. J. Nutr..

[99] McLellan B., Corder G., Giurco D., Ishihara K. (2012). Renewable energy in the minerals industry: A review of global potential. J. Clean. Prod..

[100] Kenemans J.L., Lorist M.M. (1995). Caffeine and selective visual processing. Pharmacol. Biochem. Behav..

[101] Wesensten N.J., Killgore W.D., Balkin T.J. (2005). Performance and alertness effects of caffeine, dextroamphetamine, and modafinil during sleep deprivation. J. Sleep Res..

[102] Vital-Lopez F.G., Doty T.J., Reifman J. (2024). When to sleep and consume caffeine to boost alertness. Sleep.

[103] Smith A. (2002). Effects of caffeine on human behavior. Food Chem. Toxicol..

[104] Lieberman H.R. (2001). The effects of ginseng, ephedrine, and caffeine on cognitive performance, mood and energy. Nutr. Rev..

[105] Brunyé T.T., Mahoney C.R., Lieberman H.R., Giles G.E., Taylor H.A. (2010). Acute caffeine consumption enhances the executive control of visual attention in habitual consumers. Brain Cogn..

[106] Clark W.T., Lawson M., Garnto G., Smith E., Massey K. (2023). Effect of Caffeine in Pattern Memory and Reaction Time Test Among College Students. Int. Undergrad. J. Health Sci..

[107] McLellan T.M., Caldwell J.A., Lieberman H.R. (2016). A review of caffeine’s effects on cognitive, physical and occupational performance. Neurosci. Biobehav. Rev..

[108] Tieges Z., Snel J., Kok A., Wijnen J.G., Lorist M.M., Ridderinkhof K.R. (2006). Caffeine improves anticipatory processes in task switching. Biol. Psychol..

[109] Lorist M.M., Snel J. (1997). Caffeine effects on perceptual and motor processes. Electroencephalogr. Clin. Neurophysiol..

[110] Nehlig A. (2010). Is caffeine a cognitive enhancer?. J. Alzheimer’s Dis..

[111] LaRocca E.B. (2020). The Correlation Between Personal Stressors, Anxiety and Caffeine Consumption Among JMU Faculty.

[112] McCarthy C.E., Candelario D.M., Liu M.T. (2014). Anxiety-inducing dietary supplements: A review of herbs and other supplements with anxiogenic properties. Pharmacol. Pharm..

[113] Yerkes R.M., Dodson J.D. (1908). The relation of strength of stimulus to rapidity of habit-formation. J. Comp. Neurol. Psychol..

[114] Garrett B.E., Griffiths R.R. (1997). The role of dopamine in the behavioral effects of caffeine in animals and humans. Pharmacol. Biochem. Behav..

[115] Daly J.W., Shi D., Nikodijević O., Jacobson K.A. (2020). The role of adenosine receptors in the central action of caffeine. Caffeine and Behavior: Current Views & Research Trends.

[116] Popoli P., Reggio R., Pèzzola A., Fuxe K., Ferré S. (1998). Adenosine A1 and A2A receptor antagonists stimulate motor activity: Evidence for an increased effectiveness in aged rats. Neurosci. Lett..

[117] Blanchard J., Sawers S. (1983). The absolute bioavailability of caffeine in man. Eur. J. Clin. Pharmacol..

[118] Landolt H.-P. (2008). Sleep homeostasis: A role for adenosine in humans?. Biochem. Pharmacol..

[119] Reichert C.F., Deboer T., Landolt H.P. (2022). Adenosine, caffeine, and sleep–wake regulation: State of the science and perspectives. J. Sleep Res..

[120] Sanchez S.E., Martinez C., Oriol R.A., Yanez D., Castañeda B., Sanchez E., Gelaye B., Williams M.A. (2013). Sleep quality, sleep patterns and consumption of energy drinks and other caffeinated beverages among Peruvian college students. Health.

[121] Landolt H.P., Dijk D.-J., Gaus S.E., Borbély A.A. (1995). Caffeine reduces low-frequency delta activity in the human sleep EEG. Neuropsychopharmacology.

[122] Snel J., Lorist M.M. (2011). Effects of caffeine on sleep and cognition. Prog. Brain Res..

[123] Reyner L., Horne J.A. (2002). Efficacy of a ‘functional energy drink’in counteracting driver sleepiness. Physiol. Behav..

[124] Alford C., Bhatti J., Leigh T., Jamieson A., Hindmarch I. (1996). Caffeine-induced sleep disruption: Effects on waking the following day and its reversal with an hypnotic. Hum. Psychopharmacol. Clin. Exp..

[125] Segu A., Kannan N.N. (2023). The duration of caffeine treatment plays an essential role in its effect on sleep and circadian rhythm. Sleep Adv..

[126] ACOG Committee (2010). Opinion No. 462: Moderate caffeine consumption during pregnancy. Obs. Gynecol..

[127] Román-Gálvez M.R., Martín-Peláez S., Hernández-Martínez L., Cano-Ibáñez N., Olmedo-Requena R., Martínez-Galiano J.M., Bueno-Cavanillas A., Amezcua-Prieto C. (2022). Caffeine Intake throughout Pregnancy, and Factors Associated with Non-Compliance with Recommendations: A Cohort Study. Nutrients.

[128] Fernandes O., Sabharwal M., Smiley T., Pastuszak A., Koren G., Einarson T. (1998). Moderate to heavy caffeine consumption during pregnancy and relationship to spontaneous abortion and abnormal fetal growth: A meta-analysis. Reprod. Toxicol..

[129] Rohweder R., de Oliveira Schmalfuss T., Dos Santos Borniger D., Ferreira C.Z., Zanardini M.K., Lopes G., Barbosa C.P., Moreira T.D., Schuler-Faccini L., Sanseverino M.T.V. (2024). Caffeine intake during pregnancy and adverse outcomes: An integrative review. Reprod. Toxicol..

[130] Salvador H.S., Koos B.J. (1989). Effects of regular and decaffeinated coffee on fetal breathing and heart rate. Am. J. Obs. Gynecol..

[131] Mulder E.J., Tegaldo L., Bruschettini P., Visser G.H. (2010). Foetal response to maternal coffee intake: Role of habitual versus non-habitual caffeine consumption. J. Psychopharmacol..

[132] Buscicchio G., Piemontese M., Gentilucci L., Ferretti F., Tranquilli A.L. (2012). The effects of maternal caffeine and chocolate intake on fetal heart rate. J. Matern. Fetal Neonatal Med..

[133] Lakin H., Sheehan P., Soti V. (2023). Maternal Caffeine Consumption and Its Impact on the Fetus: A Review. Cureus.

[134] Li J., Zhao H., Song J.M., Zhang J., Tang Y.L., Xin C.M. (2015). A meta-analysis of risk of pregnancy loss and caffeine and coffee consumption during pregnancy. Int. J. Gynaecol. Obstet..

[135] Jafari A., Naghshi S., Shahinfar H., Salehi S.O., Kiany F., Askari M., Surkan P.J., Azadbakht L. (2022). Relationship between maternal caffeine and coffee intake and pregnancy loss: A grading of recommendations assessment, development, and evaluation-assessed, dose-response meta-analysis of observational studies. Front. Nutr..

[136] Alsunni A.A. (2015). Energy Drink Consumption: Beneficial and Adverse Health Effects. Int. J. Health Sci..

[137] Ding M., Markon A.O., Jones-Dominic O.E., Purdue-Smithe A.C., Rich-Edwards J.W., Wolpert B.J., Chavarro J.E. (2023). Intake of Energy Drinks Before and During Pregnancy and Adverse Pregnancy Outcomes. JAMA Netw. Open.

[138] Shah S.A., Chu B.W., Lacey C.S., Riddock I.C., Lee M., Dargush A.E. (2016). Impact of Acute Energy Drink Consumption on Blood Pressure Parameters: A Meta-analysis. Ann. Pharmacother..

[139] Nawrot P., Jordan S., Eastwood J., Rotstein J., Hugenholtz A., Feeley M. (2003). Effects of caffeine on human health. Food Addit. Contam..

[140] Turnbull D., Rodricks J.V., Mariano G.F., Chowdhury F. (2017). Caffeine and cardiovascular health. Regul. Toxicol. Pharmacol..

[141] Nadeem I.M., Shanmugaraj A., Sakha S., Horner N.S., Ayeni O.R., Khan M. (2021). Energy drinks and their adverse health effects: A systematic review and meta-analysis. Sports Health.

[142] Gualberto P.I.B., Benvindo V.V., Waclawovsky G., Deresz L.F. (2024). Acute effects of energy drink consumption on cardiovascular parameters in healthy adults: A systematic review and meta-analysis of randomized clinical trials. Nutr. Rev..

[143] Elitok A., Oz F., Panc C., Sarikaya R., Sezikli S., Pala Y., Bugan O.S., Ates M., Parildar H., Ayaz M.B. (2015). Acute effects of Red Bull energy drink on ventricular repolarization in healthy young volunteers: A prospective study. Anatol. J. Cardiol..

[144] Fletcher E.A., Lacey C.S., Aaron M., Kolasa M., Occiano A., Shah S.A. (2017). Randomized Controlled Trial of High-Volume Energy Drink Versus Caffeine Consumption on ECG and Hemodynamic Parameters. J. Am. Heart Assoc..

[145] Tzang C.C., Lin W.C., Lin L.H., Lin T.Y., Chang K.V., Wu W.T., Ozcakar L. (2024). Insights into the cardiovascular benefits of taurine: A systematic review and meta-analysis. Nutr. J..

[146] Ahmadian M., Dabidi Roshan V., Ashourpore E. (2017). Taurine Supplementation Improves Functional Capacity, Myocardial Oxygen Consumption, and Electrical Activity in Heart Failure. J. Diet. Suppl..

[147] Ahmadian M., Roshan V.D., Aslani E., Stannard S.R. (2017). Taurine supplementation has anti-atherogenic and anti-inflammatory effects before and after incremental exercise in heart failure. Ther. Adv. Cardiovasc. Dis..

[148] Woolf K., Manore M.M. (2006). B-vitamins and exercise: Does exercise alter requirements?. Int. J. Sport Nutr. Exerc. Metab..

[149] Miao Y., Guo Y., Chen Y., Lin Y., Lu Y., Guo Q. (2023). The effect of B-vitamins on the prevention and treatment of cardiovascular diseases: A systematic review and meta-analysis. Nutr. Rev..

[150] Meyer K., Ball P. (2004). Psychological and Cardiovascular Effects of Guaraná and Yerba Mate: A Comparison with Coffee. Rev. Interam. Psicol..

[151] Shah S.A., Occiano A., Nguyen T.A., Chan A., Sky J.C., Bhattacharyya M., O’Dell K.M., Shek A., Nguyen N.N. (2016). Electrocardiographic and blood pressure effects of energy drinks and Panax ginseng in healthy volunteers: A randomized clinical trial. Int. J. Cardiol..

[152] Sayed W.M. (2021). Quercetin Alleviates Red Bull Energy Drink-Induced Cerebral Cortex Neurotoxicity via Modulation of Nrf2 and HO-1. Oxid. Med. Cell Longev..

[153] Alia A.O., Petrunich-Rutherford M.L. (2019). Anxiety-like behavior and whole-body cortisol responses to components of energy drinks in zebrafish (Danio rerio). PeerJ.

[154] Biggio F., Talani G., Asuni G.P., Bassareo V., Boi M., Dazzi L., Pisu M.G., Porcu P., Sanna E., Sanna F. (2024). Mixing energy drinks and alcohol during adolescence impairs brain function: A study of rat hippocampal plasticity. Neuropharmacology.

[155] Graneri L., Lam V., D’Alonzo Z., Nesbit M., Mamo J.C.L., Takechi R. (2021). The Consumption of Energy Drinks Induces Blood-Brain Barrier Dysfunction in Wild-Type Mice. Front. Nutr..

[156] Kamar S.A., Malak H.W.A., Saad S.A. (2020). Effect of caffeinated energy drinks on the structure of hippocampal cornu ammonis 1 and dentate gyrus of adult male albino rats. Anat. Cell Biol..

[157] Al-Basher G.I., Aljabal H., Almeer R.S., Allam A.A., Mahmoud A.M. (2018). Perinatal exposure to energy drink induces oxidative damage in the liver, kidney and brain, and behavioral alterations in mice offspring. Biomed. Pharmacother..

[158] Hammond D., Reid J.L., Zukowski S. (2018). Adverse effects of caffeinated energy drinks among youth and young adults in Canada: A Web-based survey. CMAJ Open.

[159] Seifert S.M., Seifert S.A., Schaechter J.L., Bronstein A.C., Benson B.E., Hershorin E.R., Arheart K.L., Franco V.I., Lipshultz S.E. (2013). An analysis of energy-drink toxicity in the National Poison Data System. Clin. Toxicol..

[160] Jackson D.A., Cotter B.V., Merchant R.C., Babu K.M., Baird J.R., Nirenberg T., Linakis J.G. (2013). Behavioral and physiologic adverse effects in adolescent and young adult emergency department patients reporting use of energy drinks and caffeine. Clin. Toxicol..

[161] Monnard C.R., Montani J.P., Grasser E.K. (2016). Cerebro- and Cardio-vascular Responses to Energy Drink in Young Adults: Is there a Gender Effect?. Front. Physiol..

[162] Curry K., Stasio M.J. (2009). The effects of energy drinks alone and with alcohol on neuropsychological functioning. Hum. Psychopharmacol..

[163] Harris J.L., Munsell C.R. (2015). Energy drinks and adolescents: What’s the harm?. Nutr. Rev..

[164] Gunja N., Brown J.A. (2012). Energy drinks: Health risks and toxicity. Med. J. Aust..

[165] Richards G., Smith A.P. (2016). A Review of Energy Drinks and Mental Health, with a Focus on Stress, Anxiety, and Depression. J. Caffeine Res..

[166] Wolk B.J., Ganetsky M., Babu K.M. (2012). Toxicity of energy drinks. Curr. Opin. Pediatr..

[167] Specterman M., Bhuiya A., Kuppuswamy A., Strutton P.H., Catley M., Davey N.J. (2005). The effect of an energy drink containing glucose and caffeine on human corticospinal excitability. Physiol. Behav..

[168] Ludwig I.A., Mena P., Calani L., Cid C., Del Rio D., Lean M.E., Crozier A. (2014). Variations in caffeine and chlorogenic acid contents of coffees: What are we drinking?. Food Funct..

[169] Sherman W.M., Brodowicz G., Wright D.A., Allen W.K., Simonsen J., Dernbach A. (1989). Effects of 4 h preexercise carbohydrate feedings on cycling performance. Med. Sci. Sports Exerc..

[170] Azagba S., Langille D., Asbridge M. (2014). An emerging adolescent health risk: Caffeinated energy drink consumption patterns among high school students. Prev. Med..

[171] Rai S.P., Ansari A.H., Singh D., Singh S. (2024). Coffee, antioxidants, and brain inflammation. Prog. Brain Res..

[172] Miller K.E., Dermen K.H., Lucke J.F. (2018). Caffeinated energy drink use by US adolescents aged 13–17: A national profile. Psychol. Addict. Behav..

[173] Ajibo C., Van Griethuysen A., Visram S., Lake A. (2024). Consumption of energy drinks by children and young people: A systematic review examining evidence of physical effects and consumer attitudes. Public Health.

[174] Pound C.M., Blair B. (2017). Energy and sports drinks in children and adolescents. Paediatr. Child. Health.

[175] Seifert S.M., Schaechter J.L., Hershorin E.R., Lipshultz S.E. (2011). Health effects of energy drinks on children, adolescents, and young adults. Pediatrics.

[176] Kim S.Y., Sim S., Choi H.G. (2017). High stress, lack of sleep, low school performance, and suicide attempts are associated with high energy drink intake in adolescents. PLoS ONE.

[177] Holubcikova J., Kolarcik P., Madarasova Geckova A., Reijneveld S.A., van Dijk J.P. (2017). Regular energy drink consumption is associated with the risk of health and behavioural problems in adolescents. Eur. J. Pediatr..

[178] Sampasa-Kanyinga H., Hamilton H.A., Chaput J.-P. (2018). Sleep duration and consumption of sugar-sweetened beverages and energy drinks among adolescents. Nutrition.

[179] Reissig C.J., Strain E.C., Griffiths R.R. (2009). Caffeinated energy drinks—A growing problem. Drug Alcohol. Depend..

[180] Temple J.L. (2009). Caffeine use in children: What we know, what we have left to learn, and why we should worry. Neurosci. Biobehav. Rev..

[181] Li P., Mandilaras G., Jakob A., Dalla-Pozza R., Haas N.A., Oberhoffer F.S. (2022). Energy drinks and their acute effects on arterial stiffness in healthy children and teenagers: A randomized trial. J. Clin. Med..

[182] Park S., Lee Y., Lee J.H. (2016). Association between energy drink intake, sleep, stress, and suicidality in Korean adolescents: Energy drink use in isolation or in combination with junk food consumption. Nutr. J..

[183] Owens J.A., Mindell J., Baylor A. (2014). Effect of energy drink and caffeinated beverage consumption on sleep, mood, and performance in children and adolescents. Nutr. Rev..

[184] Almulla A.A., Zoubeidi T. (2022). Association of overweight, obesity and insufficient sleep duration and related lifestyle factors among school children and adolescents. Int. J. Adolesc. Med. Health.

[185] Oberhoffer F.S., Dalla-Pozza R., Jakob A., Haas N.A., Mandilaras G., Li P. (2023). Energy drinks: Effects on pediatric 24-h ambulatory blood pressure monitoring. A randomized trial. Pediatr. Res..

[186] Ragsdale F.R., Gronli T.D., Batool N., Haight N., Mehaffey A., McMahon E.C., Nalli T.W., Mannello C.M., Sell C.J., McCann P.J. (2010). Effect of Red Bull energy drink on cardiovascular and renal function. Amino Acids.

[187] Kaldenbach S., Hysing M., Strand T.A., Sivertsen B. (2024). Energy drink consumption and sleep parameters in college and university students: A national cross-sectional study. BMJ Open.

[188] Caliskan S.G., Kilic M.A., Bilgin M.D. (2021). Acute effects of energy drink on hemodynamic and electrophysiologic parameters in habitual and non-habitual caffeine consumers. Clin. Nutr. ESPEN.

[189] Shah S.A., Szeto A.H., Farewell R., Shek A., Fan D., Quach K.N., Bhattacharyya M., Elmiari J., Chan W., O’Dell K. (2019). Impact of high volume energy drink consumption on electrocardiographic and blood pressure parameters: A randomized trial. J. Am. Heart Assoc..

[190] Juliano L.M., Griffiths R.R. (2004). A critical review of caffeine withdrawal: Empirical validation of symptoms and signs, incidence, severity, and associated features. Psychopharmacology.

[191] Leal W.E., Jackson D.B. (2018). Energy drinks and escalation in drug use severity: An emergent hazard to adolescent health. Prev. Med..

[192] Beer C.P.D. (2001). Caffeine: The forgotten variable. Int. J. Psychiatry Clin. Pract..

[193] Galimov A., Hanewinkel R., Hansen J., Unger J.B., Sussman S., Morgenstern M. (2020). Association of energy drink consumption with substance-use initiation among adolescents: A 12-month longitudinal study. J. Psychopharmacol..

[194] Richards G., Smith A. (2015). Caffeine consumption and self-assessed stress, anxiety, and depression in secondary school children. J. Psychopharmacol..

[195] Steinke L., Lanfear D.E., Dhanapal V., Kalus J.S. (2009). Effect of “energy drink” consumption on hemodynamic and electrocardiographic parameters in healthy young adults. Ann. Pharmacother..

[196] Kurtz A.M., Leong J., Anand M., Dargush A.E., Shah S.A. (2013). Effects of caffeinated versus decaffeinated energy shots on blood pressure and heart rate in healthy young volunteers. Pharmacother. J. Hum. Pharmacol. Drug Ther..

[197] Lee C. (2019). The Effect of Energy Drinks on Cardiovascular Variables: A Randomized Controlled Trial. Master Thesis.

[198] Basrai M., Schweinlin A., Menzel J., Mielke H., Weikert C., Dusemund B., Putze K., Watzl B., Lampen A., Bischoff S.C. (2019). Energy drinks induce acute cardiovascular and metabolic changes pointing to potential risks for young adults: A randomized controlled trial. J. Nutr..

[199] Banks N.F., Rogers E.M., Helwig N.J., Schwager L.E., Alpers J.P., Schulte S.L., Trachta E.R., Lockwood C.M., Jenkins N.D. (2024). Acute effects of commercial energy drink consumption on exercise performance and cardiovascular safety: A randomized, double-blind, placebo-controlled, crossover trial. J. Int. Soc. Sports Nutr..

[200] Svatikova A., Covassin N., Somers K.R., Somers K.V., Soucek F., Kara T., Bukartyk J. (2015). A randomized trial of cardiovascular responses to energy drink consumption in healthy adults. JAMA.

[201] Greene E., Oman K., Lefler M. (2014). Energy drink–induced acute kidney injury. Ann. Pharmacother..

[202] Chua K.Y., Li H., Lim W.-S., Koh W.-P. (2023). Consumption of coffee, tea, and caffeine at midlife, and the risk of physical frailty in late life. J. Am. Med. Dir. Assoc..

[203] Arria A.M., Caldeira K.M., Kasperski S.J., O’Grady K.E., Vincent K.B., Griffiths R.R., Wish E.D. (2010). Increased alcohol consumption, nonmedical prescription drug use, and illicit drug use are associated with energy drink consumption among college students. J. Addict. Med..

[204] Schuttler D., Hamm W., Kellnar A., Brunner S., Stremmel C. (2022). Comparable Analysis of Acute Changes in Vascular Tone after Coffee versus Energy Drink Consumption. Nutrients.

[205] Brothers R.M., Christmas K.M., Patik J.C., Bhella P.S. (2017). Heart rate, blood pressure and repolarization effects of an energy drink as compared to coffee. Clin. Physiol. Funct. Imaging.

[206] Kaur A., Yousuf H., Ramgobin-Marshall D., Jain R., Jain R. (2022). Energy drink consumption: A rising public health issue. Rev. Cardiovasc. Med..

[207] Wilson C. Rockstar Debuts Energy Drink for Improved Focus. https://www.foodbusinessnews.net/articles/25458-rockstar-debuts-energy-drink-for-improved-focus.

[208] Lowery L.M., Anderson D.E., Scanlon K.F., Stack A., Escalante G., Campbell S.C., Kerksick C.M., Nelson M.T., Ziegenfuss T.N., VanDusseldorp T.A. (2023). International society of sports nutrition position stand: Coffee and sports performance. J. Int. Soc. Sports Nutr..

[209] Socała K., Szopa A., Serefko A., Poleszak E., Wlaź P. (2020). Neuroprotective effects of coffee bioactive compounds: A review. Int. J. Mol. Sci..

[210] Reyes C.M., Cornelis M.C. (2018). Caffeine in the diet: Country-level consumption and guidelines. Nutrients.

[211] Beverages, Coffee, Brewed, Prepared with Tap Water. https://fdc.nal.usda.gov/fdc-app.html#/food-details/171890/nutrients.

[212] McCusker R.R., Goldberger B.A., Cone E.J. (2003). Caffeine content of specialty coffees. J. Anal. Toxicol..

[213] Chieng D., Canovas R., Segan L., Sugumar H., Voskoboinik A., Prabhu S., Ling L.-H., Lee G., Morton J.B., Kaye D.M. (2022). The impact of coffee subtypes on incident cardiovascular disease, arrhythmias, and mortality: Long-term outcomes from the UK Biobank. Eur. J. Prev. Cardiol..

[214] Nieber K. (2017). The impact of coffee on health. Planta Medica.

[215] Surma S., Romańczyk M., Filipiak K.J., Lip G.Y. (2023). Coffee and cardiac arrhythmias: Up-date review of the literature and clinical studies. Cardiol. J..

[216] Jagim A.R., Harty P.S., Fischer K.M., Kerksick C.M., Erickson J.L. (2020). Adverse events reported to the United States Food and Drug Administration related to caffeine-containing products. Mayo Clin. Proc..

